# Identification and verification of aging-related lncRNAs for prognosis prediction and immune microenvironment in patients with head and neck squamous carcinoma

**DOI:** 10.32604/or.2022.028193

**Published:** 2023-03-01

**Authors:** QING GAO, YUJING SHI, YUANYUAN SUN, SHU ZHOU, ZEYUAN LIU, XINCHEN SUN, XIAOKE DI

**Affiliations:** 1 Department of Radiation Oncology, The First Affiliated Hospital of Nanjing Medical University, Nanjing, 210029, China; 2 Department of Oncology, Jurong People’s Hospital, Jurong, 212499, China; 3 Department of Radiation Oncology, Nanjing Jiangning Hospital and the Affiliated Jiangning Hospital of Nanjing Medical University, Nanjing, 211199, China

**Keywords:** Aging, lncRNA, HNSCC, Prognosis, Tumor immune microenvironment, Bioinformatics

## Abstract

Aging is highly associated with tumor formation and progression. However, little research has explored the association of aging-related lncRNAs (ARLs) with the prognosis and tumor immune microenvironment (TIME) of head and neck squamous cell carcinoma (HNSCC). RNA sequences and clinicopathological data of HNSCC patients and normal subjects were downloaded from The Cancer Genome Atlas. In the training group, we used Pearson correlation, univariate Cox regression, least absolute shrinkage/selection operator regression analyses, and multivariate Cox regression to build a prognostic model. In the test group, we evaluated the model. Multivariate Cox regression was done to screen out independent prognostic factors, with which we constructed a nomogram. Afterward, we demonstrated the predictive value of the risk scores based on the model and the nomogram using time-dependent receiver operating characteristics. Gene set enrichment analysis, immune correlation analysis, and half-maximal inhibitory concentration were also performed to reveal the different landscapes of TIME between risk groups and to predict immuno- and chemo-therapeutic responses. The most important LINC00861 in the model was examined in HNE1, CNE1, and CNE2 nasopharyngeal carcinoma cell lines and transfected into the cell lines CNE1 and CNE2 using the LINC00861-pcDNA3.1 construct plasmid. In addition, CCK-8, Edu, and SA-β-gal staining assays were conducted to test the biofunction of LINC00861 in the CNE1 and CNE2 cells. The signature based on nine ARLs has a good predictive value in survival time, immune infiltration, immune checkpoint expression, and sensitivity to multiple drugs. LINC00861 expression in CNE2 was significantly lower than in the HNE1 and CNE1 cells, and LINC00861 overexpression significantly inhibited the proliferation and increased the senescence of nasopharyngeal carcinoma cell lines. This work built and verified a new prognostic model for HNSCC based on ARLs and mapped the immune landscape in HNSCC. LINC00861 is a protective factor for the development of HNSCC.

## Introduction

Head and neck squamous cell carcinoma (HNSCC) is a type of malignancy originating from the mucosal epithelium of the lip, oral cavity, larynx, or naso-, oro-, hypo-pharynx. HNSCC has a mortality rate of nearly 50% in the first five years after diagnosis, with over 850,000 new cases and more than 400,000 deaths globally in 2020 [1–3]. Though diverse treatment modalities such as surgery, targeted therapy, chemo-, radio- and immuno-therapy are used in HNSCC patients, their clinical benefits are far from satisfactory [4].

Aging is an irreversible growth arrest condition closely associated with the development of many chronic diseases and cancers [5]. Cellular senescence can be induced by stimuli such as telomere shortening due to extensive replication, oxidative stress, DNA damage, and oncogene overexpression, which are regulated by senescence-related genes [6]. After senescent cells lose proliferative capacity, hindering tumor progression, they remain viable and metabolically active, secreting various cytokines that promote the growth, migration, and invasion of tumor cells [7,8]. The potential prognostic value of aging-related genes in HNSCC and the correlations with inflammation and tumor immunity have recently been explored and confirmed [9].

Long non-coding RNAs (lncRNAs) are a class of non-protein-coding RNAs that are more than 200 nucleotides in length and have specific functions (e.g., splicing, transcriptional and post-transcriptional modulation of mRNA), which are largely linked to tumor development, metastasis, and tumor immunity [10]. The action mechanisms of lncRNAs in aging have not yet been elucidated, and the prognostic impact of lncRNAs associated with aging-related genes in HNSCC remains unknown.

Here, we use bioinformatics to probe into the mechanisms of aging-related lncRNAs (ARLs) in HNSCC, identify some new molecular biomarkers linked with prognosis, and build an effective prognostic model to forecast the survival of HNSCC patients and function as their new treatment target.

## Materials and Methods

### Data collection

We downloaded the RNA sequencing data and clinical data of HNSCC and normal tissues from The Cancer Genome Atlas (TCGA) (https://portal.gdc.cancer.gov/). Aging-related genes were acquired from the Human Ageing Genomic Resources (HAGR; Table S1). Expression data were standardized to fragment per kilobase million. Based on Strawberry Perl, we integrated and processed the data, distinguished between lncRNA and mRNA, extracted complete clinical information, and removed data missing survival status and unknown survival time or <30 days.

### Identification of ARLs in HNSCC

We performed co-expression analysis of lncRNAs and AGs in tumor samples (correlation coefficient > 0.5 and *p* < 0.001) with packages Strawberry Perl and limma R and mapped a network with igraph R. Differential expression of ARLs in cancer *versus* normal tissue samples was analyzed (Log2 fold change (FC) > 1, false discovery rate (FDR) < 0.05, *p* < 0.05) [11], and a heatmap was plotted with the R package pheatmap.

### Construction of the Prognostic Signature

Based on the clinical data of HNSCC, lncRNAs with significant effects on OS were screened from ARLs (*p* < 0.05) using univariate Cox analysis. Samples were randomly and evenly separated into a training risk group and a test risk group on the R package caret. In the training group, we conducted the least absolute shrinkage and selection operator (LASSO) Cox regression on the R package glmnet and multivariate Cox analysis to recognize the ARLs for risk models and computed the risk score as follows:

$$\begin{array}{cc} \text{Risk score =} & {\text{Expression of the 1}}^{\text{st}}\text{ lncRNA * coefficient}\\ & {\text{+Expression of the 2}}^{\text{nd}}\text{ lncRNA * coefficient}\\ & \text{+Expression of the nth lncRNA * coefficient} \end{array}\;$$

where the coefficient denoted the regression coefficient of the corresponding lncRNA [12]. The overall group was classified by the median risk score into low- and high-risk groups.

### Verification of risk signature and establishment of nomogram

We performed the Kaplan–Meier survival curve and log-rank test on the R package survminer to clarify whether the low-risk group has longer OS than the high-risk group. The 1, 3- and 5-year survival receiver’s operating characteristic (ROC) curves and the areas under the curves (AUCs) were conducted on the R package timeROC to assess the prediction efficacy in comparison with other clinical data [13]. Based on the R package rms, a nomogram for forecasting survival was built on basis of the risk score and clinical features, which were detected by both uni-/multi-variate Cox regression. We assessed the accuracy of the nomogram via calibration curve and ROC analysis.

### Gene set enrichment analyses (GSEA) and assessment of the immune landscape

We performed GSEA (version 4.2.3) using the curated gene set (kegg.v7.5.1 symbols.gmt), screened at *p* < 0.05 and FDR < 0.25, to determine the different biofunctions and pathways between the two groups [14]. Immune cell infiltration files for HNSCC in the TCGA were obtained from UCSC Xena (https://xena.ucsc.edu/). Pearson correlation analyses between risk score and immune cell infiltration scores were conducted by the R packages “limma”, “scales”, “ggplot2”, “reshape2”, “tidyverse”, “ggpubr” and “ggtext” and we screened out and visualized the results at *p* < 0.05. The TME scores of HNSCC were calculated using the ESTIMATE algorithm with estimate R package. Then we assessed immune cell infiltration and immune cell function by single-sample GSEA (ssGSEA), and we accessed immune checkpoint activity by comparing differential expression of immune checkpoint genes.

### Prediction of drug therapy response

We adopted the Genomics of Cancer Drug Sensitivity in Cancer (https://www.cancerrxgene.org) and R package pRRophetic to forecast the sensitivity to drug therapies as per the half-maximal inhibitory concentration (IC50) in the two groups. All the code files mentioned above can be downloaded and used at the following website: https://github.com/yexian123/ARLs-HNSCC-analysis.git.

### Cell culture and cell transfection

We purchased three human nasopharyngeal carcinoma cell lines (HNE1, CNE1, and CNE2) from the Chinese Academy of Sciences (Shanghai, China). All cells were grown in an RPMI-1640 medium (KeyGEN, Nanjing, China) with 10% fetal bovine serum (FBS, PAN-Seratech, Germany) and 1% penicillin/streptomycin in 5% CO_2_ at 37°C. The pcDNA3.1 and LINC00861-pcDNA3.1 bought from Genescript Biotechnology were transfected into CNE1 and CNE2 cells using Lipotransfectamine 3000 (Thermo). After 48 h, the CNE1 and CNE2 cells were harvested.

### Quantitative real-time PCR (qRT-PCR)

Total RNA from each cell line was extracted with an RNA-easy isolation reagent, and reversed with HiScript® III RT SuperMix for qPCR (+gDNA wiper) (both Vazyme Biotech, China) to synthesize complementary DNA (cDNA). The PCR system was made from SYBR Green® Premix Ex Taq™ (Vazyme Biotech, China). Results of individual lncRNAs were standardized to the GAPDH expression. The primer sequences for LINC00861 and GAPDH are listed in Table S2.

### Cell counting kit-8 (CCK-8) assay

Cell proliferation was detected using CCK-8 assay (Beyotime, Shanghai, China) according to the instructions. Cells (1000/well) were planted into 96-well plates and grown in an RPMI-1640 medium containing 10% FBS. At the same time each day, 10 μl of a CCK-8 solution was added to each well, and further incubated for 2 h at 37°C. Absorbance at 450 nm was detected using a microplate spectrophotometer (Thermo, USA) and used to estimate the proliferative capacity of CNE1 and CNE2 cells.

### EdU assay

We assessed cell proliferation using an EdU assay kit (RiboBio, China). CNE1 and CNE2 cells (5 × 10^4^ cells/well) were inoculated in a confocal laser cuvette, then cultured in a medium with 50 μM EdU (C10310-1, RiboBio) for 2 h and treated as per the instructions. Images were obtained under a fluorescent microscope. The mean proportion of EdU-positive cells in three random fields of view was analyzed.

### Senescence-associated β-galactosidase (SA-β-gal) staining

Cellular SA-β-gal activity were assayed according to the manufacturer’s instructions (C0602, Beyotime, Nanjing, China). CNE1 and CNE2 cells were fixed and stained with the fixative solution and staining solution mixture provided in the kit. Observe and photograph the cells under an ordinary light microscope. The number of SA-β-gal-positive cells in 3 to 5 regions of the 6-well plate was randomly counted as a percentage of the total number of cells, and the mean value was calculated.

### Statistical analysis

All statistical analysis and graphical visualization were conducted on R software 4.1.2. Categorical data between groups were compared via the chi-square test. Continuous variables in normal distribution (containing risk score and TME scores) were compared between two or more groups via Student’s *t*-test or one-way ANOVA. Differences in expression levels between immune checkpoint genes and ARLs were investigated via Wilcox test. The significance level was probability less than 0.05.

## Results

### Identification of aging-related LncRNAs in HNSCC patients

**Figure 1:**
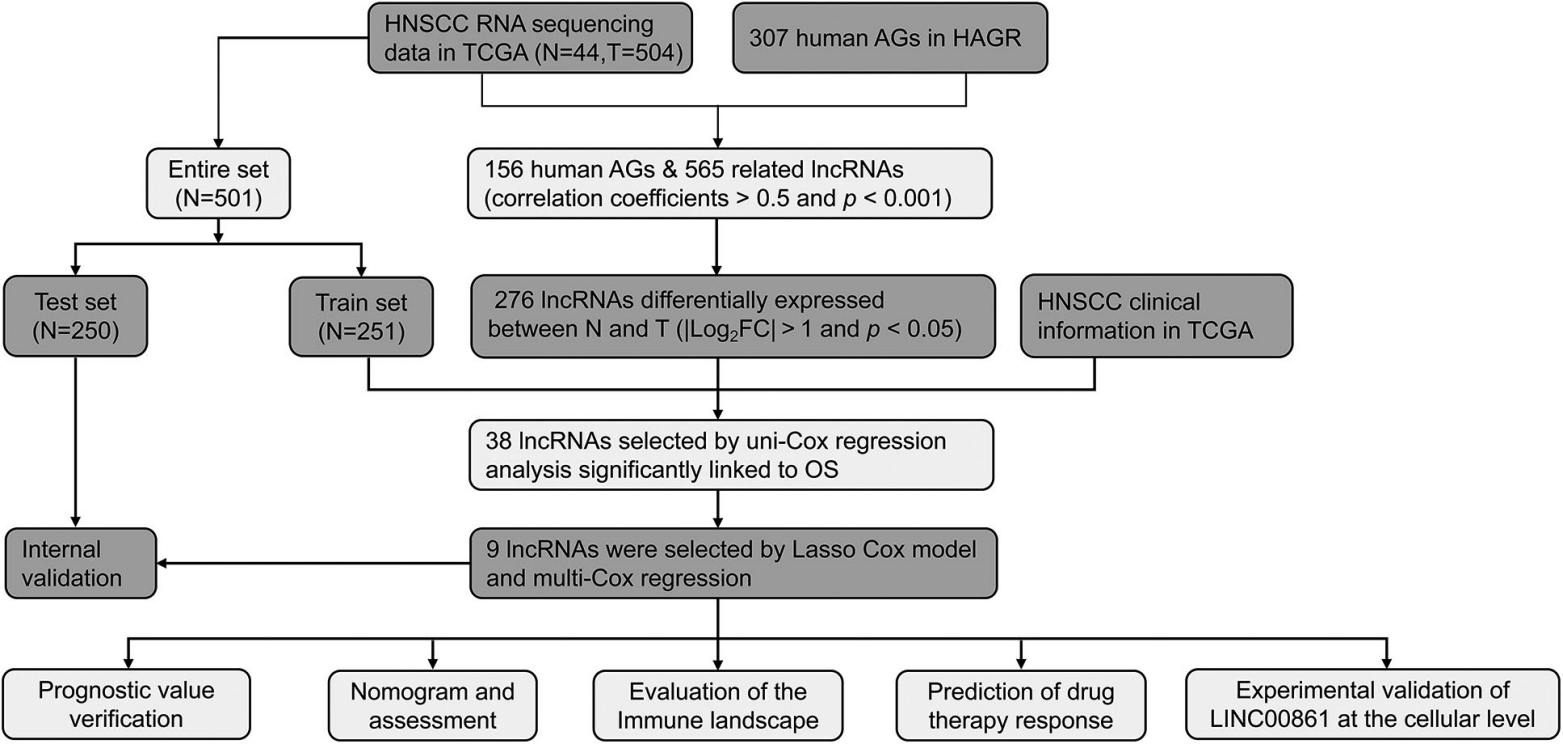
The flow chart of the whole analysis process.

Fig. 1 illustrates the flowchart of the study. Firstly, we downloaded transcript data of 44 normal and 504 cancer samples from TCGA on 2022-04-15. Then 307 aging-related genes (ARGs) were obtained from HAGR (Table S1). After that, we got 156 AGs and 565 ALRs by correlation analysis (correlation coefficient > 0.5 and *p* < 0.001).

**Figure 2:**
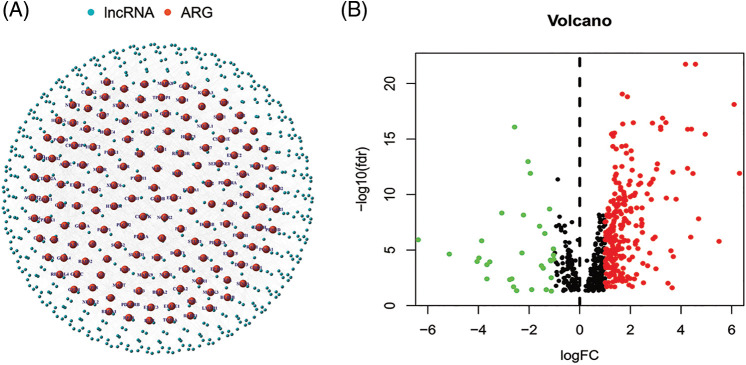
(A) The regulatory network showed 565 lncRNAs associated with 156 AGs. (B) The 276 significantly different ALRs in cancer versus normal tissue samples.

The regulatory network of these lncRNAs with AGs is shown in Fig. 2A. Based on differential expression analysis of the ALRs in cancer *versus* normal tissue samples (|Log_2_FC| > 1 and *p* < 0.05), we acquired 276 significantly different ALRs, of which 244 were up-regulated and 32 were down-regulated in cancer tissues (Table S3, Fig. 2B).

### Establishment and verification of the prognostic model

**Figure 3:**
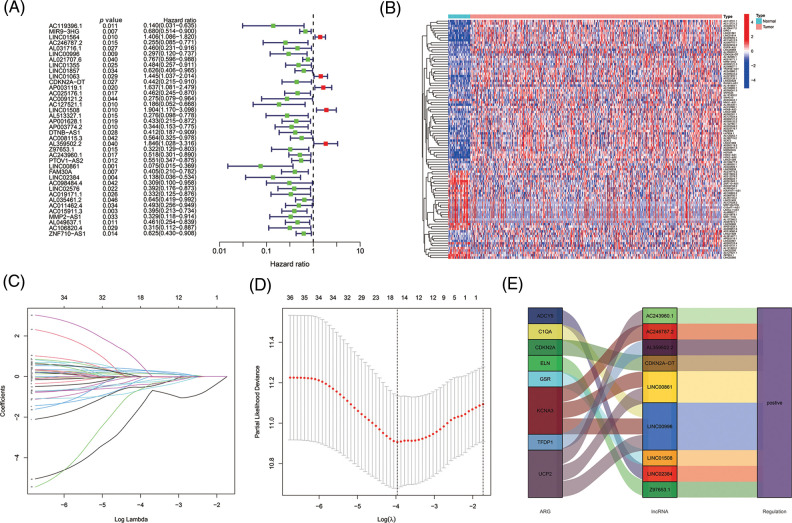
Selecting eligible ARLs in the training group for building the model. (A) ARLs affecting OS extracted by univariate Cox regression analysis. (B) The heatmap showed the expression profiles of 38 prognostic lncRNAs. (C) The 10-fold cross-validation for variable selection in the LASSO model. (D) The LASSO coefficient profile of 16 ARLs, the intersection of the middle dashed line with the upper X-axis is the number of lncRNAs obtained from the LASSO regression, and the intersection with the lower X-axis is the value of Log(λ). (E) The Sankey diagram of 8 aging-related genes and 9 associated lncRNAs.

The 501 eligible tumor samples with complete information were equally divided into a train (N = 251) group and a test group (N = 250). In the training group, univariate Cox regression analysis identified 38 ALRs significantly related with overall survival (OS) (*p* < 0.05) (Figs. 3A and 3B). To avoid over-fitting of the prognostic features, we used LASSO regression of these lncRNAs and isolated sixteen lncRNAs associated with AGs in HNSCC when the first-order value of Log(λ) was the smallest likelihood of bias (Figs. 3C and 3D), where Log(λ) was −3.96. After that, we performed multi-COX regression analysis and finalized nine ARLs used to construct the model. The Sankey diagram (Fig. 3E) demonstrated that these nine lncRNAs were positively associated with the AGs ADCY5, C1QA, CDKN2A, ELN, GSR, KCNA3, TFDP1 and UCP2.

Risk scores were calculated as follows: AC246787.2 × (−1.08765834515961) + LINC00996 × (−1.00608978255594) +`CDKN2A-DT` × (−0.754633772241435) + LINC01508 × (0.524759230799357) + AL359502.2 × (0.64309513483616) + Z97653.1 × (−1.22717339963094) + AC243960.1 × (3.02676937595734) + LINC00861 × (−4.95297301646684) + LINC02384 × (−1.88205538422933).

**Figure 4:**
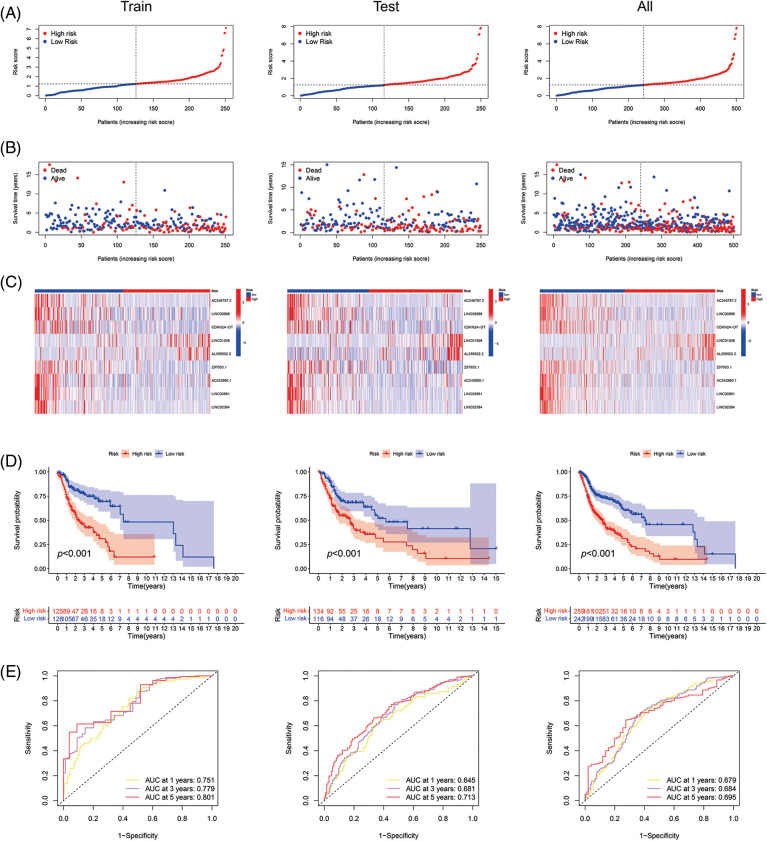

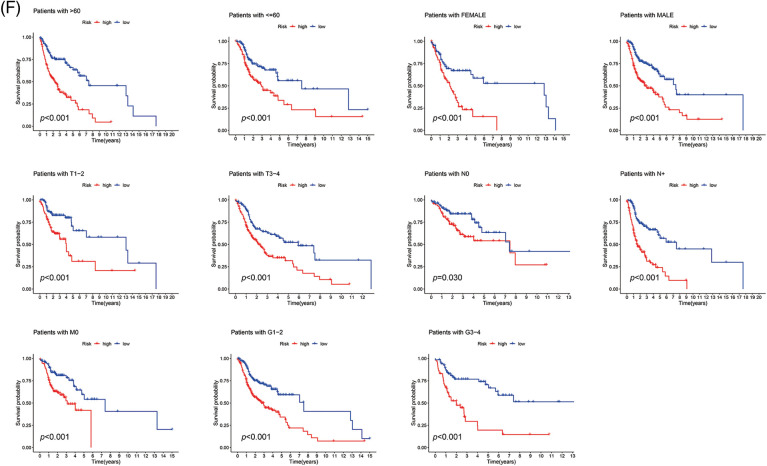
Prognosis value of the 9 ARLs model in the train, test, and all sets. (A) Exhibition of ARLs model based on risk score of the train, test, and entire sets, respectively. (B) Survival time and survival status between low- and high-risk groups in the train, test, and all sets, respectively. (C) The heat map of 9 ARLs expressions in the train, test, and all sets, respectively. (D) Kaplan-Meier survival curve of OS for patients between risk groups in the train, test, and all sets, respectively. (E) The train, test, and all sets’ 1-, 3-, and 5-year ROC curves, respectively. (F) Kaplan-Meier survival analysis for HNSCC patients stratified by age, gender, T, N, or M staging, and grade in all set.

With the risk score formula, we divided the train, test, and entire groups into low- and high-risk groups. We compared the distribution of risk scores, survival status, lncRNA expression, and survival time among the risk groups. All results showed significant differences between the low- and high-risk groups (Figs. 4A–4D). Time-dependent receiver operating characteristics (ROC) were used to assess the sensitivity and specificity of the model for prognosis. We also illustrated the ROC results in terms of the area under the ROC curve (AUC); the 1-, 3- and 5-year AUCs were 0.751, 0.779, and 0.801 for the train set, 0.645, 0.681, and 0.713 for the test set and 0.679, 0.684 and 0.695 for the whole group, respectively (Fig. 4E). In the entire set, patients showed the same results for age, gender, T, N, or M staging, and grade (Fig. 4F).

### Independent prognostic ability of the signature

**Figure 5:**
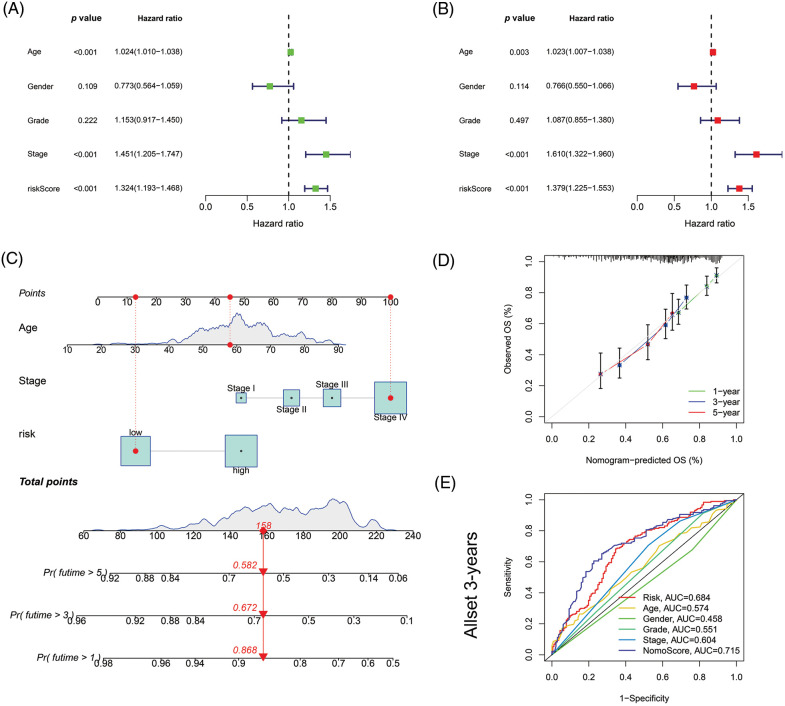
Nomogram and assessment of the risk model. (A) Uni-Cox and (B) multi-Cox analyses of clinical factors and risk score with OS. (C) The nomogram that integrated the age, tumor stage, and risk score predicted the probability of the 1-, 3-, and 5-year OS. (D) The calibration curves for 1-, 3-, and 5-year OS. (E) The 3-year ROC curves of risk score, nomogram total score, and clinical characteristics.

In univariate Cox regression analyses for all tumor samples, the hazard ratio (HR) for the risk score was 1.324, with a 95% confidence interval (CI) of 1.193–1.468 (*p* < 0.001). In addition, age, and stage were also statistically different (Fig. 5A). In the multivariate Cox regression, risk score (HR: 1.379, 95% CI: 1.225–1.553, *p* < 0.001), age (HR: 1.023, 95% CI: 1.007–1.038; *p* = 0.003) and stage (HR: 1.610, 95% CI: 1.322–1.960; *p* < 0.001) were independent prognostic factors of HNSCC (Fig. 5B).

Then based on risk score, age, and stage, we constructed a nomogram to predict the incidence rates of 1-, 3-, and 5-year OS in HNSCC patients (Fig. 5C). The calibration curves demonstrated a high concordance between the actual and nomogram-predicted survival rates of HNSCC patients (Fig. 5D). The sensitivity and specificity of the model for prognosis were evaluated via time-dependent ROC. The risk score (AUC = 0.684) and nomogram score (AUC = 0.715) had better predictive ability than age, gender, grade, and stage in the 3-year ROC of the risk model (Fig. 5E).

### Gene set enrichment analyses and investigation of immune status

**Figure 6:**
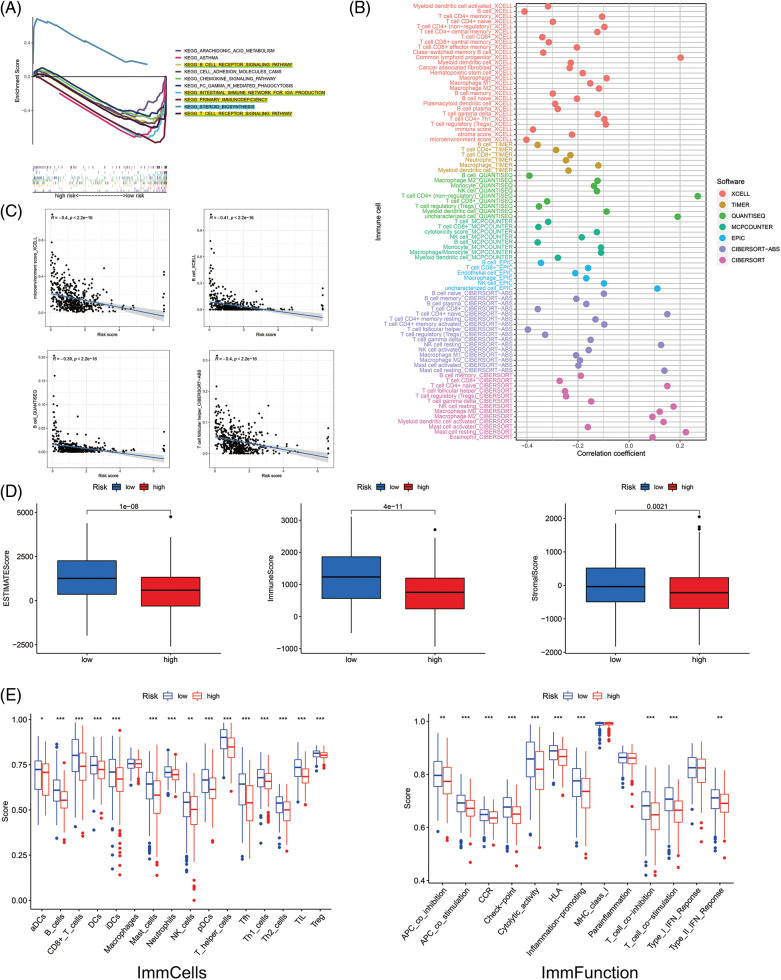
The investigation of tumor immune factors and immunotherapy. (A) GSEA of the top 10 pathways significantly enriched in the low- and high-risk groups. (B) The immune cell bubble of risk groups. (C) The correlation between risk score and immune cells. (D) The comparison of immune-related scores between low- and high-risk groups. (E) The difference between immune cell infiltration and immune functions in risk groups. *p < 0.05; **p < 0.01; ***p < 0.001.

Firstly, with the GSEA software, we explored the KEGG pathways in low- and high-risk groups to investigate the biofunction differences. Of the top 10 significantly enriched pathways, the high-risk group was enriched in only one pathway related to steroid biosynthesis. In comparison, the low-risk group was enriched in four immune-associated KEGG pathways (all FDR < 0.25) (Fig. 6A). Then we analyzed the relevance of immune cells to the low- and high-risk groups using data from multiple platforms. Results showed the majority of immune cells were related to the low-risk group, which generally agrees with the GSEA results (Fig. 6B). The content of B cells, follicular helper T cells (cTfh), was negatively related to the risk score, suggesting they were more abundant in the low-risk group (Fig. 6C), which had larger immune score and ESTIMATE score (Fig. 6D). Furthermore, the low-risk group had more immune cell infiltration such as B cells, CD8+ T cells, and dendritic cells (DCs), and more prosperous immune functions (Fig. 6E). The above results imply that the low-risk group is under a higher immune infiltration state.

### Clinical treatment response analyses

**Figure 7:**
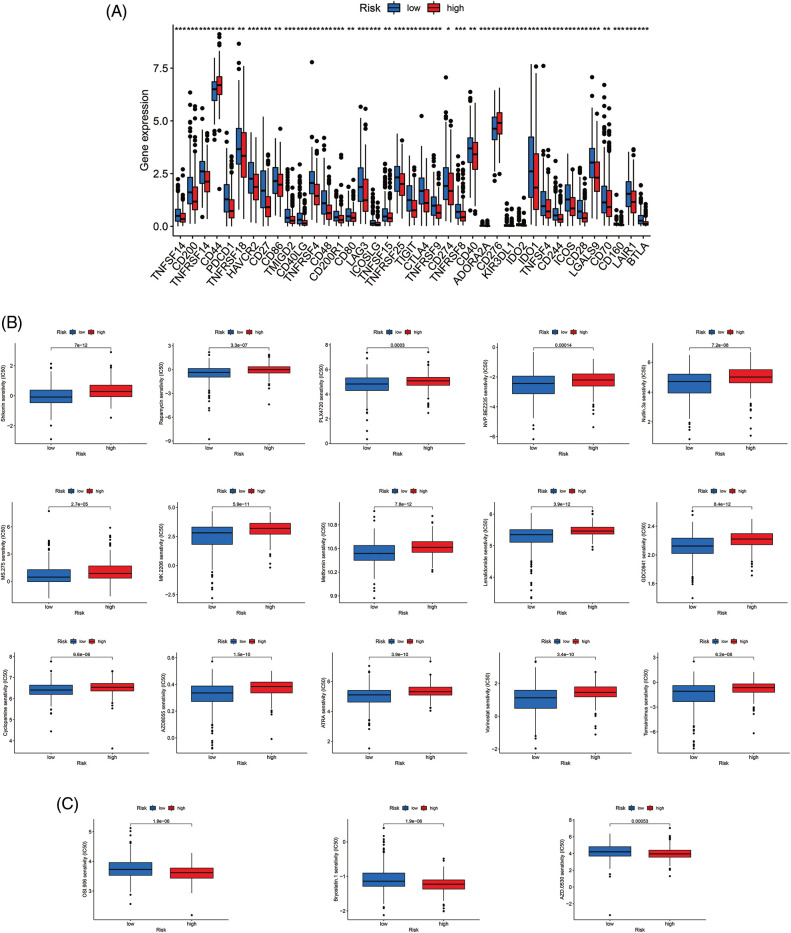
Immune checkpoints and drug sensitivity analyses. (A) The difference in immune checkpoint expression in risk groups. (B) Fifteen immunotherapeutic drugs showed lower IC50 values in the low-risk group. (C) Three immunotherapeutic drugs showed lower IC50 values in the high-risk group. *p < 0.05; **p < 0.01; ***p < 0.001.

**Figure S1:**
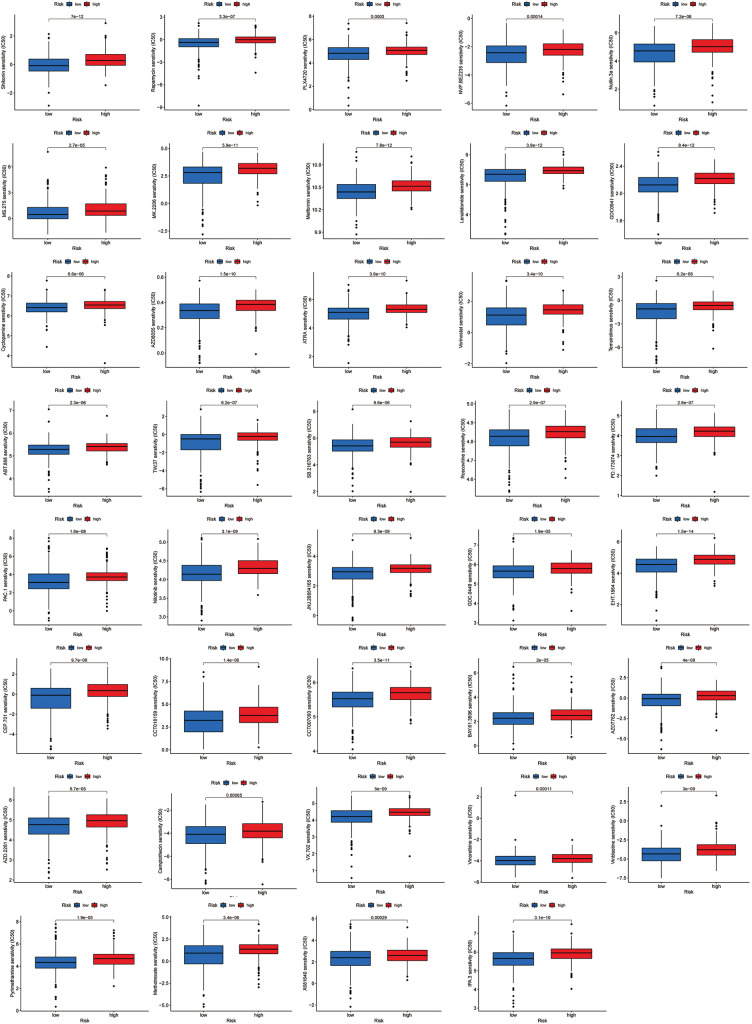
All 39 chemicals with lower IC50 values in the low-risk group.

**Figure S2:**
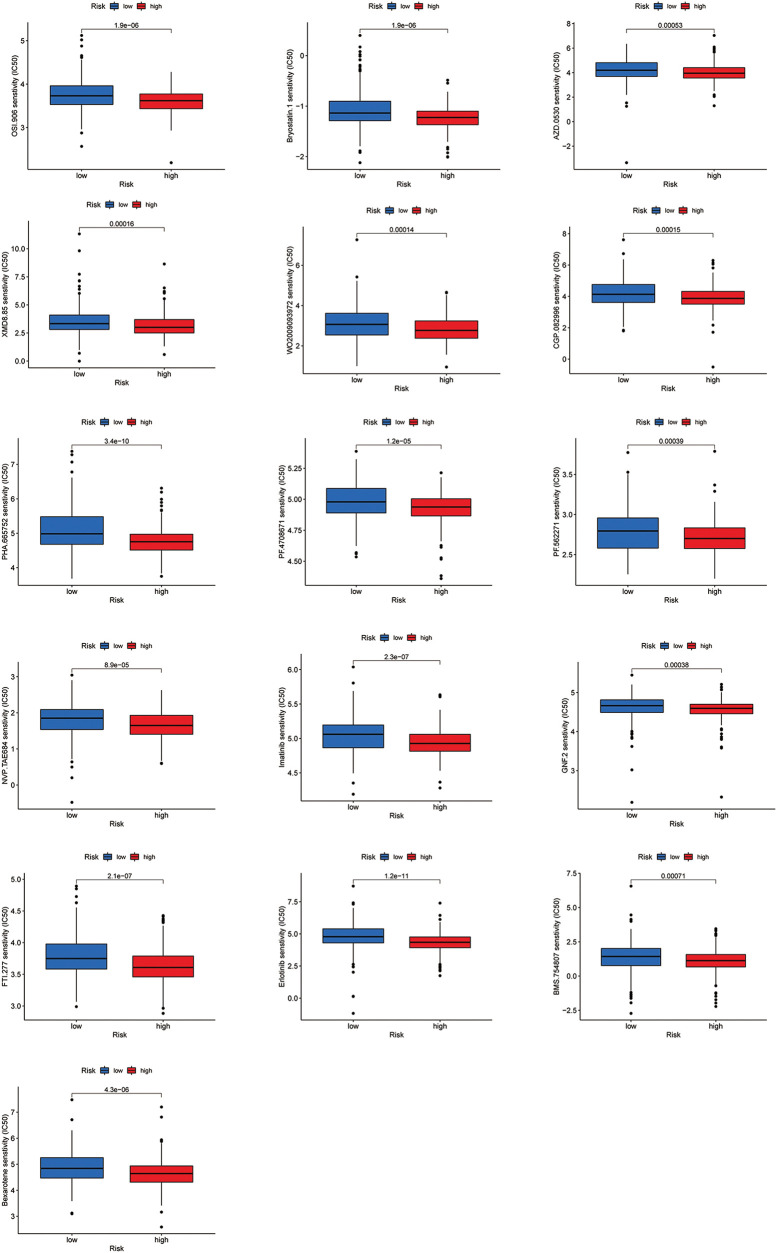
All 16 chemicals with lower IC50 values in the high-risk group.

Most immune checkpoints were also better activated in the low-risk group (Fig. 7A), which means we can group HNSCC patients by the risk pattern to select the appropriate checkpoint inhibitor. The low-risk group with higher immune scores had smaller IC50 in 15 immunotherapeutic agents (Fig. 7B) [15–29] and was more sensitive to 18 targeted therapeutics (e.g., Nilotinib) and six chemotherapeutic agents (e.g., Vinblastine) than the high-risk group. All 39 chemicals were shown in Fig. S1. Sixteen drugs had lower IC50 in the high-risk group (Fig. S2), of which three drugs including Bryostatin.1 were associated with immunotherapy (Fig. 7C) [30–32].

### LINC00861 inhibited the proliferation of nasopharyngeal carcinoma cells

**Figure 8:**
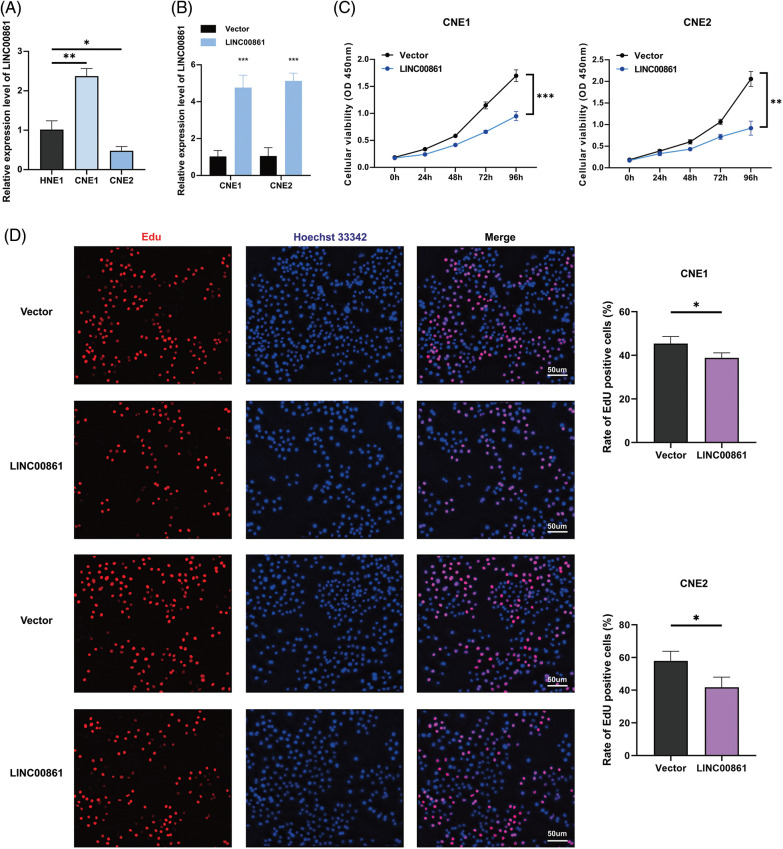

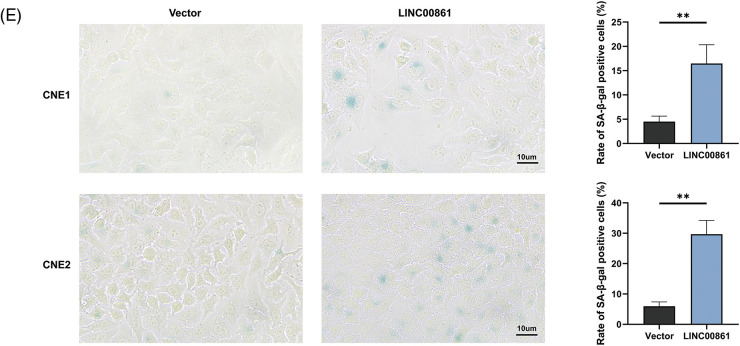
Cellular experiments. (A) and (B) Expression level of LINC00861. (C) CCK-8, (D) Edu, and (E) SA-β-gal staining assay in CNE1 and CNE2 cell lines in different treatment groups. *p < 0.05; **p < 0.01; ***p < 0.001.

We selected LINC00861 for further analysis since it had the highest correlation with the risk signature. The qRT-PCR indicated that LINC00861 was differentially expressed in three different nasopharyngeal carcinoma cell lines (Fig. 8A). We transfected the LINC00861-PCDNA3.1 plasmid in CNE1 and CNE2 cells to overexpress it and verified the transfection efficiency by qRT-PCR (Fig. 8B). CCK-8, Edu, and SA-β-gal Staining assays showed that LINC00861 overexpression considerably inhibited the growth and increased the senescence of CNE1 and CNE2 cells (Figs. 8C–8E).

## Discussion

Surgery, radiotherapy, and standard chemotherapy are commonly used for HNSCC. Recently, medicine including cetuximab, an anti-EGFR, and pembrolizumab, an anti-PD-1 therapy, was accepted for late-stage, recurrent, or metastatic HNSCC [33,34]. Regardless of therapeutic progress, the 5-year OS rate of HNSCC is still low (<50%) due to its ease of invasion, metastasis, development of chemo-resistance, and the fact that most cases are diagnosed at late stages [35]. Moreover, the 3-year OS rate is around 80% for HPV+ HNSCC and 55% for HPV- HNSCC [36]. Recently, a variety of novel biomarkers such as PITX2 methylation and C1GALT1 autoantibody that forecast prognosis, and B7-H3 that predicts immunotherapeutic prognosis and reaction in HNSCC were identified gradually [37–39]. However, the prognostic models and biomarkers need to be updated since more new drugs, including ICP inhibitors, are put into clinical practice. Here, a new model based on ARLs was constructed for the first time in HNSCC, which can both predict prognosis and differentiate the TIME.

Aging is closely connected with the occurrence of many chronic diseases and tumors [5]. Cell senescence is a physiological process whereby cells lose the proliferative ability forever. Cell senescence is triggered by various stimuli and the DNA damage response (DDR), leading to stimulation of the p53 and/or p16INK4A pathways, and its important regulatory factors include the cell cycle regulators p53, p21, p16, and pRb proteins [40]. The increased activity of lysosomal senescence-related β-galactosidase (SA-β-Gal) is an important marker of senescent cells [41]. Cell senescence is a double-edged sword in malignant tumors, as it prevents tumor development and promotes HNSCC progression through the secretion of various factors, called senescence-associated secretory phenotype (SASP). Notch, mTOR, and NF-κB pathways are engaged in SASP management [42]. The majority of studies conclude that cellular senescence acts primarily as a cancer suppressor rather than a cancer promoter [43–50]. Silencing peroxiredoxin1(Prx1)-induced cellular senescence blocks malignant conversion and inhibits the formation of oral squamous carcinoma [51]. In nasopharyngeal carcinoma, senescent cells promote tumor cell invasion by increasing the secretion of matrix metalloproteinase 9 (MMP 9) [52]. Bioinformatics analyses reveal the critical function of AGs in predicting the survival prognosis and revealing the TIME landscape of HNSCC [9,53].

Recently, many studies show that lncRNAs are critical in HNSCC occurrence, and can both promote and suppress cancer. More importantly, some lncRNAs can serve as biomarkers and treatment targets for HNSCC and can predict patient prognosis [2,54,55]. However, as the significant member involved in transcriptional and post-transcriptional regulation, the adjusting role of aging by lncRNAs in HNSCC is unknown. Therefore, we analytically validated the link of lncRNAs with aging and the prognosis of HNSCC, aiming to make some contribution to subsequent studies.

By LASSO and multi-cox regression analysis, we identified nine lncRNAs associated with eight AGs in HNSCC, all of which were positively regulated with the genes (Fig. 3E). Three lncRNAs (LINC01508, AL359502.2, AC243960.1) were related with a high risk of patients with poor prognosis, and the remaining 6 lncRNAs (AC246787.2, LINC00996, `CDKN2A-DT`, Z97653.1, LINC00861, LINC02384) were associated with low risk. These nine lncRNAs were enrolled into a prediction model. Through validation, the model was proved to be reliable and stable (Figs. 4, 5A–5E). By comparing with published prognostic models of HNSCC constructed based on ferroptosis-related lncRNAs, we found that these articles had high AUC predictive values for 3-year survival (0.83, 0.811, 0.726, and 0.687, respectively) [56–59], the 3-year AUC in our results was 0.779 for the train set, so collectively it seems that our proposed model shows a good accuracy in predicting prognosis. Reportedly, LINC01508 upexpression inhibited cisplatin tolerance of ovarian cancer cells by suppressing the Hippo-YAP pathway [60]. In cervical cancer cells, LINC00861 inhibits tumor progression and EMT by acting as a ceRNA for miR-513b-5p and modulating the PTEN/AKT/mTOR pathway [61]. The biological functions of the rest lncRNAs used in the modeling are unknown, about which researchers can carry out future experiments.

Aging not only regulates cell proliferation, but also alters the TIME through the secretion of SASP, which contains MMP, growth factors (VEGF and GM-CSF), inflammatory factors (IL-6 and IL-8), and the accumulation of lipofuscin deposits [62]. GSEA implied that the low-risk group was enriched with immune pathways such as the B cell receptor (BCR) and T cell receptor (TCR) pathways, and had higher immune infiltration and immune score, which may be related to their better prognosis (Figs. 6A and 6D). Considering the function of cell senescence in regulating TME, we conducted ssGSEA to explore the immune state in the low- and high-risk groups. The immune cells (aDCs, B cells, CD8+ T cells, DCs, iDCs, mast cells, neutrophils, NK cells, pDCs, T helper cells, Tfh cells, Th1 cells, Th2 cells, TIL, Tregs) and immune roles (APC (or T cell) co-suppression, APC (or T cell) co-stimulation, CCR, checkpoint, cytolytic activity, HLA, proinflammation, type II IFN response) were more active in the low-risk group (Figs. 6B and 6E). ICPs including PD-1, CTLA4, CD200, CD27, CD86, CD80, CD70, and TIGIT were more expressed in the low-risk group (Fig. 7A). These results imply that we can use the risk scores of ARLs to well distinguish the immune infiltration status of HNSCC and which is higher in the low-risk group. SASP factors released by senescent cells recruit innate immune cells (neutrophils, NK cells) and adaptive immune cells (CD8+ T cells) to modulate the removal of senescent tumor cells [63]. Therefore, the low-risk group may have more active cell senescence and thus is can be more cured by immunotherapy.

Radio-, chemo-, immuno-, and targeted therapies can induce cellular senescence and play an important role in TME through SASP [64]. Most HNSCC patients receive radiotherapy, which alters TME and activates the immune response of tumor cells [65,66] and may account for better outcomes in the low-risk group. As for drug sensitivity, the low-risk group was more sensitive to six inhibitors of the PI3K/AKT/mTOR pathway, such as rapamycin, which not only enhanced immunotherapeutic effects [18,22–25,29] but also blocked SASP-induced tumor progression (Fig. 7B). One study found that rapamycin prevented radiation-induced secretion of NFκB-driven pro-inflammatory SASP from prostate cancer cells and fibroblasts, thereby inhibiting tumor progression [67]. As a result, our study can guide clinical treatment and provide evidence for improving future immunotherapy and seeking appropriate target populations. Moreover, LINC00861 was identified as a protective factor against HNSCC progression *in vitro* (Fig. 8).

However, our study still has some limitations. Firstly, both our training and validation sets were from TCGA, which may cause bias, so the results may be more plausible if external validation was performed. Secondly, we only performed qRT-PCR, CCK-8, Edu, and SA-β-gal staining assays validation of LINC00861 in cell lines, but did not do further functional experiments or tissue validation, so further experiments are still needed. Finally, more clinical follow-up data shall be used to demonstrate the value of our prognostic model.

In conclusion, we built a robust prognostic prediction model for HNSCC using nine ARLs. It has a good predictive value in survival time, immune infiltration, immune checkpoint expression, and sensitivity to multiple drugs. LINC00861 acts as a protective factor against HNSCC progression.

## Data Availability

The public datasets to advocate our results can be gained from TCGA (https://portal.gdc.cancer.gov).

## Appendix

**TABLE S1:** The list of aging-related genes

Serial number	Gene	Entrez gene id
1	GHR	2690
2	GHRH	2691
3	SHC1	6464
4	POU1F1	5449
5	PROP1	5626
6	TP53	7157
7	TERC	7012
8	TERT	7015
9	ATM	472
10	PLAU	5328
11	ERCC2	2068
12	ERCC8	1161
13	WRN	7486
14	LMNA	4000
15	IGF1R	3480
16	TXN	7295
17	KL	9365
18	E2F1	1869
19	PTPN11	5781
20	NFKB2	4791
21	STAT5B	6777
22	STAT3	6774
23	STAT5A	6776
24	NRG1	3084
25	HDAC3	8841
26	GH1	2688
27	IL7R	3575
28	IGF1	3479
29	IGF2	3481
30	INS	3630
31	NGF	4803
32	IRS1	3667
33	PTPN1	5770
34	IRS2	8660
35	AKT1	207
36	PIK3CB	5291
37	NGFR	4804
38	HRAS	3265
39	MYC	4609
40	EGFR	1956
41	ERBB2	2064
42	INSR	3643
43	NCOR1	9611
44	NBN	4683
45	JUND	3727
46	IL2	3558
47	PDGFB	5155
48	EGF	1950
49	IL2RG	3561
50	FOS	2353
51	PDGFRB	5159
52	EPOR	2057
53	SST	6750
54	PRKCD	5580
55	PPARA	5465
56	RET	5979
57	PLCG2	5336
58	PEX5	5830
59	TCF3	6929
60	PARP1	142
61	BRCA1	672
62	PIN1	5300
63	PTEN	5728
64	CREBBP	1387
65	HIF1A	3091
66	UBB	7314
67	RPA1	6117
68	BLM	641
69	BCL2	596
70	S100B	6285
71	VCP	7415
72	POLG	5428
73	IGFBP3	3486
74	HSP90AA1	3320
75	NR3C1	2908
76	EGR1	1958
77	VEGFA	7422
78	ABL1	25
79	BRCA2	675
80	TOP2A	7153
81	TOP2B	7155
82	NFKB1	4790
83	TOP1	7150
84	RAD51	5888
85	UBE2I	7329
86	TNF	7124
87	PDPK1	5170
88	CEBPA	1050
89	CEBPB	1051
90	MXI1	4601
91	TGFB1	7040
92	ERCC6	2074
93	STK11	6794
94	EP300	2033
95	APTX	54840
96	PML	5371
97	GSK3B	2932
98	HTT	3064
99	PRKCA	5578
100	SSTR3	6753
101	HELLS	3070
102	APOC3	345
103	EEF2	1938
104	ERCC3	2071
105	TERF1	7013
106	PRKDC	5591
107	CAT	847
108	ERCC5	2073
109	AR	367
110	GTF2H2	2966
111	XRCC5	7520
112	PCNA	5111
113	FEN1	2237
114	FAS	355
115	TERF2	7014
116	XRCC6	2547
117	POLD1	5424
118	BAX	581
119	RB1	5925
120	EMD	2010
121	GRB2	2885
122	FOXO3	2309
123	FOXO1	2308
124	HSF1	3297
125	XPA	7507
126	MSRA	4482
127	RECQL4	9401
128	SOD2	6648
129	SOD1	6647
130	FOXM1	2305
131	COQ7	10229
132	CACNA1A	773
133	LRP2	4036
134	AIFM1	9131
135	UCHL1	7345
136	APP	351
137	APOE	348
138	A2M	2
139	SNCG	6623
140	PRDX1	5052
141	PON1	5444
142	RELA	5970
143	IL6	3569
144	RGN	9104
145	ATP5O	539
146	RAD52	5893
147	TOP3B	8940
148	ERCC1	2067
149	SIRT1	23411
150	HDAC1	3065
151	HSPA9	3313
152	GPX1	2876
153	GSR	2936
154	GSS	2937
155	GSTA4	2941
156	GSTP1	2950
157	MT-CO1	4512
158	HSPD1	3329
159	HSPA1A	3303
160	HSPA1B	3304
161	PCMT1	5110
162	MAPK8	5599
163	YWHAZ	7534
164	PTK2B	2185
165	PTK2	5747
166	IL7	3574
167	MAPK14	1432
168	FGFR1	2260
169	SP1	6667
170	FLT1	2321
171	JUN	3725
172	MED1	5469
173	MAPK9	5601
174	MAPK3	5595
175	HMGB1	3146
176	CCNA2	890
177	HMGB2	3148
178	MAP3K5	4217
179	TAF1	6872
180	LMNB1	4001
181	SDHC	6391
182	FOXO4	4303
183	HESX1	8820
184	PIK3R1	5295
185	BSCL2	26580
186	AGPAT2	10555
187	BMI1	648
188	EEF1A1	1915
189	TFAP2A	7020
190	BDNF	627
191	CREB1	1385
192	ATF2	1386
193	TBP	6908
194	APEX1	328
195	HBP1	26959
196	BUB1B	701
197	PTGS2	5743
198	HSPA8	3312
199	SIN3A	25942
200	CDK1	983
201	TFDP1	7027
202	DDIT3	1649
203	POLA1	5422
204	MAPT	4137
205	CTGF	1490
206	HDAC2	3066
207	MAX	4149
208	MXD1	4084
209	MDM2	4193
210	SUMO1	7341
211	H2AFX	3014
212	HOXB7	3217
213	HOXC4	3221
214	JAK2	3717
215	ESR1	2099
216	LEP	3952
217	LEPR	3953
218	NFKBIA	4792
219	CLU	1191
220	MTOR	2475
221	GHRHR	2692
222	CTNNB1	1499
223	PSEN1	5663
224	DLL3	10683
225	CDKN2A	1029
226	PPP1CA	5499
227	DBN1	1627
228	NOG	9241
229	ELN	2006
230	ATR	545
231	UCP3	7352
232	ZMPSTE24	10269
233	TP63	8626
234	UCP2	7351
235	POLB	5423
236	GCLC	2729
237	GCLM	2730
238	SIRT6	51548
239	BUB3	9184
240	RAE1	8480
241	PMCH	5367
242	MLH1	4292
243	CSNK1E	1454
244	STUB1	10273
245	PPM1D	8493
246	CHEK2	11200
247	PCK1	5105
248	ARHGAP1	392
249	CDC42	998
250	ARNTL	406
251	CLOCK	9575
252	HIC1	3090
253	PAPPA	5069
254	ADCY5	111
255	PPARGC1A	10891
256	GPX4	2879
257	UCP1	7350
258	FGF23	8074
259	EFEMP1	2202
260	ERCC4	2072
261	CETP	1071
262	PPARG	5468
263	AGTR1	185
264	CISD2	493856
265	EEF1E1	9521
266	EPS8	2059
267	KCNA3	3738
268	SIRT7	51547
269	SLC13A1	6561
270	SOCS2	8835
271	TPP2	7174
272	TP53BP1	7158
273	SIRT3	23410
274	NCOR2	9612
275	SUN1	23353
276	BAK1	578
277	IGFBP2	3485
278	PYCR1	5831
279	TP73	7161
280	CNR1	1268
281	NFE2L2	4780
282	CDKN1A	1026
283	PDGFRA	5156
284	PIK3CA	5290
285	C1QA	712
286	CDKN2B	1030
287	EIF5A2	56648
288	MIF	4282
289	DGAT1	8694
290	MT1E	4493
291	FGF21	26291
292	HTRA2	27429
293	GSK3A	2931
294	NUDT1	4521
295	IKBKB	3551
296	SQSTM1	8878
297	CDK7	1022
298	GRN	2896
299	SERPINE1	5054
300	SPRTN	83932
301	RICTOR	253260
302	CTF1	1489
303	TRAP1	10131
304	TRPV1	7442
305	NFE2L1	4779
306	IFNB1	3456
307	GDF11	10220

**TABLE S2:** The primer sequences for LINC00861 and GAPDH

Oligo name	Sequence (5′ to 3′)
LINC00861-F	CTTATTGGACTCCTTGTG
LINC00861-R	ATCAGGCATCTTAGAAGT
GAPDH-F	CTGGGCTACACTGAGCACC
GAPDH-R	AAGTGGTCGTTGAGGGCAATG

**Table S3:** The list of differentially expressed ARLs in cancer *versus* normal tissue samples

Gene	conMean	treatMean	logFC	*p* value	fdr
PRKAR1B-AS1	0.592106818	1.311415476	1.147195454	4.02E-10	2.57E-09
AC119396.1	0.059656818	0.126340278	1.082555711	0.007549119	0.00893204
LINC02561	0.064047727	0.685313492	3.419544808	2.46E-11	2.08E-10
AL022724.3	0.059529545	0.264846429	2.153478271	1.38E-11	1.24E-10
AC009097.2	0.026788636	0.123094841	2.200077252	6.71E-07	1.92E-06
AC010735.2	0.105477273	0.352538095	1.74084699	1.85E-05	3.99E-05
AC145098.1	0.183954545	0.471088889	1.35664998	4.60E-09	2.20E-08
MIR9-3HG	0.080913636	1.131265079	3.805410352	3.27E-11	2.64E-10
LINC01564	0.231195455	0.667140079	1.528876682	0.004186634	0.00535772
MELTF-AS1	0.411640909	1.46177004	1.828258099	4.32E-09	2.10E-08
AC112721.2	0.015875	0.337893651	4.411740745	3.05E-18	1.29E-16
LINC01614	0.033863636	2.317105952	6.096443303	7.01E-21	7.70E-19
HCG27	0.069168182	0.171063492	1.306351458	3.87E-07	1.22E-06
AL161669.1	0.033295455	0.18774504	2.495377651	9.00E-05	0.000158293
AL133243.2	0.326981818	0.73845119	1.175292149	1.88E-09	9.91E-09
AL354836.1	1.547475	3.476730159	1.167814996	1.44E-07	5.01E-07
HCP5	2.751772727	7.471085317	1.440956516	1.00E-13	1.41E-12
LINC02560	21.91396818	7.299477976	−1.585985553	1.73E-08	7.09E-08
LINC01089	0.435547727	0.888201587	1.028056332	3.05E-06	7.85E-06
AL590822.3	0.043784091	0.116705357	1.414392125	2.99E-05	5.90E-05
ACTN1-AS1	0.055275	0.132533532	1.261658391	5.33E-06	1.29E-05
AP003419.3	0.136920455	0.33032877	1.270564639	1.46E-08	6.22E-08
MIR503HG	0.060875	0.587624802	3.270973516	1.44E-19	1.32E-17
AL022322.1	0.1554	0.473619048	1.6077406	0.001203539	0.001716215
AC026333.4	0.103131818	0.321893651	1.64209462	2.73E-05	5.52E-05
AL596442.2	0.078036364	0.267449206	1.777046465	1.86E-11	1.62E-10
AC009093.1	0.030340909	0.222134722	2.872098702	6.03E-19	3.68E-17
AC092718.4	4.190329545	10.46430357	1.320340686	2.08E-17	5.72E-16
AC246787.2	0.03065	0.118119444	1.946287497	0.01223313	0.014020852
AC112721.1	0.012890909	0.1652	3.679787773	6.33E-14	9.93E-13
AL590550.1	0.064534091	0.810038294	3.649856723	0.02347574	0.025025594
AC026369.3	0.042506818	0.150797222	1.826843677	0.004749685	0.005926311
CHROMR	0.315825	0.818041468	1.373048602	8.28E-18	2.84E-16
AL031716.1	0.149415909	0.40905873	1.452974224	6.05E-10	3.65E-09
AC011498.6	0.162872727	0.355849405	1.127521775	4.72E-08	1.78E-07
AL121820.2	1.441647727	0.530622222	−1.441961678	0.000269053	0.000431901
AC008537.4	0.045329545	0.138539683	1.611775673	1.31E-08	5.64E-08
AC013731.1	0.056738636	0.229286706	2.014749329	2.70E-10	1.76E-09
AL499627.1	1.149647727	0.169480952	−2.761996815	0.003570615	0.004623273
AL592424.1	1.555277273	0.242740079	−2.679687565	0.002939199	0.003878895
LINC00996	0.065829545	0.212509325	1.69071901	1.65E-08	6.79E-08
AC091057.1	0.343511364	0.932531944	1.440795321	7.34E-13	8.57E-12
AC092301.1	0.063281818	0.128624603	1.02330367	4.90E-05	9.05E-05
AP001528.1	0.685377273	0.141072421	−2.280462381	7.83E-06	1.83E-05
SUGT1-DT	0.149606818	0.491792857	1.716874855	3.30E-11	2.64E-10
AC120053.1	0.693888636	1.391120238	1.003471076	6.42E-09	2.99E-08
AC072054.1	0.125393182	0.319719841	1.350349374	4.06E-06	1.01E-05
AC068700.1	0.861193182	0.10203254	−3.077307575	7.94E-10	4.64E-09
AC106900.1	0.616802273	1.255901786	1.02584366	7.38E-06	1.74E-05
AC006064.3	0.072884091	0.16945	1.217183793	0.000887857	0.001292926
AP006545.2	0.065556818	0.163561706	1.319017279	0.000441418	0.000676924
AC013486.1	0.078052273	0.318375794	2.028218105	5.26E-16	1.16E-14
ZKSCAN2-DT	0.096931818	0.325807143	1.748976015	1.04E-11	9.65E-11
AL021707.6	1.072056818	2.738724603	1.353122831	1.45E-09	7.89E-09
LINC02875	0.161656818	0.337526587	1.062066792	0.0184267	0.020151909
AC027228.2	0.129661364	0.339077579	1.386866742	9.23E-12	8.73E-11
AL121832.2	1.137827273	2.328614881	1.033190492	1.38E-07	4.84E-07
AC116351.2	0.094095455	0.238725397	1.343155118	4.73E-05	8.83E-05
LINC02195	0.322002273	1.473206349	2.193816744	7.46E-12	7.32E-11
LINC01355	0.134452273	0.437241865	1.701337401	9.57E-11	6.91E-10
LINC01857	0.180195455	0.4948875	1.457537983	2.78E-06	7.41E-06
AC011676.1	0.099188636	0.309208532	1.640333375	2.98E-08	1.17E-07
NCK1-DT	0.183425	0.484146032	1.400251984	1.94E-14	3.44E-13
AL355338.1	0.005295455	0.117448214	4.471126442	8.93E-14	1.29E-12
EML4-AS1	0.031490909	0.147599206	2.228677653	1.52E-08	6.35E-08
MIR133A1HG	2.474472727	0.069697421	−5.149872065	1.03E-05	2.30E-05
AC010883.1	0.143184091	0.303369841	1.083206466	0.000110656	0.000192858
SNHG1	4.474340909	10.55369226	1.238000734	5.68E-11	4.21E-10
AC009121.3	0.024163636	0.133040278	2.4609536	2.39E-10	1.60E-09
AC025171.5	0.099713636	0.248420238	1.316919991	5.25E-08	1.96E-07
HOTAIR	0.014118182	0.27079504	4.261575101	3.38E-18	1.33E-16
AL353135.2	0.913797727	0.245503968	−1.89612851	0.035224621	0.036418676
Z82243.1	0.227154545	0.695952976	1.615315655	2.16E-12	2.24E-11
TYMSOS	0.092736364	0.26678869	1.524490448	1.48E-06	4.06E-06
AP000251.1	0.240809091	0.781058532	1.697540809	1.55E-05	3.41E-05
AL161729.4	0.059809091	0.196772421	1.718091335	0.000270872	0.000433554
Z94721.1	0.094002273	0.231449603	1.299930546	1.54E-06	4.20E-06
AC092903.2	0.067372727	0.259241865	1.944062112	1.26E-12	1.33E-11
AC093788.1	0.117952273	0.352106349	1.577808025	5.18E-10	3.20E-09
LINC01063	0.186931818	0.592612103	1.664575936	2.83E-06	7.50E-06
AL133215.2	0.176154545	0.508850595	1.530400423	2.63E-11	2.18E-10
ACAP2-IT1	0.128834091	0.281550198	1.127877771	1.01E-05	2.28E-05
AC010331.1	0.086888636	0.217527778	1.323960228	2.07E-07	7.01E-07
AC026740.1	0.474802273	1.669662103	1.814157422	4.41E-11	3.31E-10
CDKN2A-DT	0.016793182	0.203906944	3.6019634	4.76E-06	1.16E-05
AL356417.2	0.056622727	0.269382937	2.250205325	2.36E-10	1.60E-09
AC022509.1	0.050390909	0.238436905	2.242372161	0.001081502	0.001554305
AC025265.1	0.055659091	0.147030556	1.401426754	0.000200931	0.000331265
DNM1P35	0.043734091	0.10712996	1.292531795	2.89E-07	9.62E-07
AP003119.1	0.023597727	0.151401786	2.681662398	0.000154078	0.000258681
AC145285.6	0.103611364	0.220322817	1.088436674	1.77E-09	9.42E-09
AC025176.1	0.043427273	0.36392996	3.066987567	9.20E-15	1.74E-13
AL390719.2	1.208693182	2.47210873	1.032294125	0.000250786	0.000406141
MIR4435-2HG	0.144947727	0.359086905	1.308800329	1.87E-16	4.64E-15
GAS6-DT	0.353336364	0.818673413	1.212245811	1.50E-05	3.30E-05
TBX2-AS1	0.215127273	0.483406349	1.168045988	0.001800101	0.002493444
AL049775.1	0.044809091	0.242944643	2.438764258	3.58E-09	1.77E-08
AC026401.3	5.498145455	17.61770813	1.680009279	4.89E-22	8.95E-20
AC137695.3	0.183334091	0.423179762	1.206795554	1.35E-09	7.42E-09
AC023983.2	0.057461364	0.148349603	1.368336932	0.001266512	0.001792049
AC006480.2	0.063063636	0.13145119	1.059646942	3.88E-07	1.22E-06
AC005253.1	0.080818182	0.20824623	1.365538579	3.41E-13	4.35E-12
AC020907.4	0.084809091	0.273107738	1.687179367	1.19E-08	5.22E-08
HOXC-AS2	0.016709091	0.302418452	4.177841022	6.71E-25	1.84E-22
AL109761.1	0.040688636	0.105859921	1.379458642	0.018772647	0.020397884
AC073352.1	0.045825	0.151292659	1.723135199	5.62E-07	1.65E-06
MIRLET7A1HG	0.220218182	0.605296627	1.458708725	1.97E-10	1.35E-09
LINC01943	0.131563636	0.570139087	2.115553123	4.97E-19	3.41E-17
AC068205.2	0.033270455	0.132138095	1.989732972	4.91E-13	6.00E-12
AC009121.2	0.022795455	0.144438095	2.663633218	8.99E-12	8.66E-11
PACERR	0.018888636	0.133916865	2.825747203	3.65E-11	2.82E-10
AC244090.2	0.048368182	0.128838889	1.413437912	0.004903106	0.00607631
AC022762.2	0.091440909	0.212068056	1.213615668	3.65E-05	7.08E-05
RNF139-AS1	0.065327273	0.212172817	1.69948252	8.09E-19	4.44E-17
AL132780.1	0.132520455	0.27010119	1.027284942	6.88E-08	2.52E-07
AL137246.2	6.400956818	0.077033929	−6.376649764	3.72E-07	1.19E-06
AC096992.2	0.333747727	0.758723016	1.18481529	9.46E-08	3.37E-07
AC005722.4	0.838709091	0.409424206	−1.034574093	1.27E-05	2.82E-05
AC010319.3	0.086625	0.291965278	1.752941453	5.42E-09	2.57E-08
TFAP2E-AS1	0.146131818	0.293392857	1.005563409	3.77E-07	1.20E-06
AC127521.1	0.381852273	0.146793254	−1.379228941	9.02E-08	3.24E-07
AL391427.1	0.086825	0.590102183	2.764782384	8.10E-06	1.87E-05
LINC01508	0.038218182	0.218822817	2.51743213	0.007458368	0.008862866
HOXC-AS1	0.064204545	0.589673611	3.199169289	3.78E-18	1.38E-16
AC127029.2	0.961263636	0.075820635	−3.664269716	0.000120478	0.000208016
AC034243.1	0.334618182	0.153391468	−1.125297596	0.002276244	0.003093213
AC126118.1	0.056038636	0.168192659	1.585620981	6.31E-07	1.82E-06
SNHG25	0.706902273	1.419560516	1.005861667	8.58E-06	1.97E-05
DIRC1	0.019552273	0.159981548	3.032497294	3.18E-14	5.30E-13
AL513327.1	0.084647727	0.247378175	1.547174983	8.33E-13	9.33E-12
AC009120.2	0.167290909	0.339346825	1.020401468	8.02E-06	1.86E-05
AC131159.1	0.080854545	0.241655754	1.579552561	1.61E-05	3.50E-05
DCUN1D2-AS	0.047070455	0.14439246	1.617101722	2.99E-06	7.77E-06
LINC01311	0.156152273	0.47987996	1.61972	3.64E-12	3.70E-11
AL359504.1	0.093420455	0.226084127	1.275049336	9.85E-06	2.23E-05
NFE2L1-DT	0.058636364	0.167922024	1.517923917	9.57E-11	6.91E-10
AC069224.1	0.444370455	0.216301389	−1.038719969	2.97E-06	7.75E-06
AC022126.1	0.0251	0.176782937	2.81621976	7.02E-13	8.37E-12
MIR155HG	0.192284091	0.654593849	1.767360644	2.76E-09	1.40E-08
AP001628.1	0.104309091	0.278675794	1.417722793	0.000483959	0.000738038
AC108477.2	0.17665	1.315722024	2.896889065	3.14E-13	4.11E-12
AL139123.1	0.063181818	0.21734127	1.782380787	4.38E-07	1.33E-06
AC116914.2	0.279645455	0.732472421	1.389175559	6.29E-10	3.75E-09
AC036108.3	0.281513636	0.129439484	−1.120927044	0.04990337	0.04990337
LINC00839	0.119436364	0.939369246	2.975450215	1.96E-07	6.74E-07
AL355488.1	0.204727273	0.733584921	1.84126068	4.67E-10	2.95E-09
AL031186.1	0.186995455	0.639932143	1.774915731	1.19E-10	8.36E-10
AC127024.4	0.2001	0.40564246	1.019487506	6.44E-05	0.000115177
AC093249.2	0.038977273	0.203960714	2.387586243	1.53E-13	2.06E-12
CEP83-DT	0.056068182	0.141645437	1.33702993	7.71E-09	3.47E-08
AC145207.8	0.115415909	0.271279762	1.232939322	0.017305331	0.019115949
AP003774.2	0.116634091	0.266663294	1.193029716	4.39E-06	1.08E-05
AL354993.2	0.127090909	0.311897619	1.295211703	6.07E-05	0.00011038
HOXB-AS3	0.022184091	0.109029365	2.297119412	2.74E-06	7.35E-06
DNM3OS	0.243929545	0.68033869	1.479788625	3.89E-06	9.80E-06
AC016735.1	0.110979545	0.357187103	1.686386191	0.005343516	0.006592338
H1-10-AS1	0.148163636	0.328806349	1.150046745	2.15E-05	4.52E-05
AC245140.3	0.083015909	0.185961905	1.163547363	0.000240341	0.000391535
AC138696.2	0.704263636	1.625050992	1.206297491	3.20E-08	1.24E-07
AC019080.4	0.087754545	1.136444643	3.694909746	1.80E-05	3.90E-05
DTNB-AS1	0.08195	0.19453373	1.247204471	0.001252071	0.001776193
AC008764.6	0.130602273	0.337656746	1.370377379	1.05E-10	7.51E-10
MIR924HG	0.056906818	0.163723611	1.524588971	3.62E-06	9.21E-06
AC112715.1	0.047213636	0.180911111	1.93800551	2.29E-06	6.20E-06
LINC02878	0.091475	0.328319841	1.843652523	8.97E-16	1.89E-14
AP001107.4	0.131822727	0.323122619	1.293482621	4.14E-07	1.27E-06
LINC00668	0.030245455	0.782925	4.694083864	3.04E-09	1.53E-08
AC008115.3	0.214531818	0.625257143	1.543257997	3.48E-11	2.73E-10
AL512598.1	0.299693182	0.619722817	1.048136825	0.009204981	0.010775128
AC106820.3	0.269581818	0.603508532	1.162650968	4.55E-06	1.12E-05
AL109614.1	0.227370455	0.479156944	1.075453481	2.87E-05	5.75E-05
AL669970.3	0.048590909	0.204374206	2.072454799	0.017528978	0.019324115
AL359502.2	0.002897727	0.1313	5.501801288	5.68E-07	1.65E-06
SNHG10	0.235940909	0.485372421	1.040666551	1.22E-07	4.32E-07
LINC01405	1.400861364	0.110391667	−3.665611012	0.003008614	0.003951505
AC110285.2	0.191338636	0.485860714	1.344414562	0.000146178	0.000246929
LINC01561	0.005931818	0.183959722	4.954771736	1.27E-17	3.68E-16
Z97653.1	0.840370455	0.204221627	−2.040889786	5.51E-15	1.08E-13
LINC00900	0.259986364	0.113860317	−1.191170928	3.13E-10	2.02E-09
AC008610.1	0.293952273	0.751307937	1.35382241	5.64E-07	1.65E-06
LINC02614	0.061006818	0.253286905	2.053730096	3.04E-15	6.18E-14
AC125494.3	0.034561364	0.111413294	1.688689337	0.000842145	0.001229621
LANCL1-AS1	1.182338636	0.210258929	−2.49140433	0.045009563	0.045339908
RUNX3-AS1	0.044131818	0.234130952	2.407424582	8.14E-13	9.31E-12
USP30-AS1	0.132927273	0.322709524	1.279599022	0.000213779	0.000351391
AC243960.1	0.180836364	0.36402004	1.009333062	0.006945789	0.008307708
BX322234.2	0.007402273	0.586691667	6.308490399	8.39E-14	1.25E-12
AC134312.5	0.027340909	0.649378571	4.56992666	3.97E-25	1.84E-22
AC132192.2	0.105672727	0.419791468	1.990069762	1.85E-11	1.62E-10
AC018766.1	0.041334091	0.107173413	1.374542988	0.000142357	0.000241963
AC012236.1	0.281672727	0.629433929	1.160035064	0.001314671	0.00185541
AC087239.1	0.095506818	0.229099405	1.262298075	3.81E-06	9.63E-06
AC005840.2	0.332643182	0.727333929	1.128642413	7.98E-06	1.86E-05
PTOV1-AS2	0.321547727	0.804373611	1.32283286	3.68E-08	1.41E-07
LINC00861	0.049022727	0.107516071	1.133029676	0.006924901	0.008300809
AC025171.1	0.491988636	1.206895437	1.29460379	9.84E-18	3.18E-16
AC015878.1	2.951079545	0.201547619	−3.872050163	4.92E-07	1.47E-06
AC004947.3	0.022297727	0.120975198	2.439742731	1.61E-08	6.70E-08
AC011444.1	0.130077273	0.285846032	1.135869348	3.02E-07	9.88E-07
FAM30A	0.071579545	0.231084722	1.690802594	0.002428903	0.003276334
U47924.1	0.022309091	0.12407619	2.475522667	7.11E-08	2.58E-07
AC234917.3	0.405525	0.969063492	1.256800336	1.19E-17	3.64E-16
AL162595.1	0.472193182	0.980981944	1.054849373	3.03E-06	7.85E-06
AC017083.1	0.029752273	0.126128175	2.083818799	1.33E-11	1.22E-10
EGFR-AS1	0.020063636	0.110419643	2.460341829	9.10E-09	4.03E-08
KIAA1671-AS1	1.225152273	0.262661905	−2.221682192	1.27E-09	7.05E-09
AC002116.2	0.113470455	0.342654167	1.594436533	3.31E-11	2.64E-10
LINC02384	0.045775	0.122877381	1.42458758	0.002809373	0.003734493
AC027796.4	0.302159091	0.86402877	1.515771003	1.39E-10	9.66E-10
AL442125.2	0.083547727	0.224490873	1.425984303	4.14E-07	1.27E-06
AC087392.1	0.151518182	0.451799008	1.576190179	0.002246275	0.003060062
AL021978.1	0.084397727	0.891939087	3.401669134	4.21E-19	3.30E-17
AC098484.4	0.086040909	0.22107877	1.361465817	7.95E-10	4.64E-09
AC004148.1	0.286656818	0.649252183	1.179454365	5.42E-06	1.30E-05
DLEU2	0.100020455	0.271358333	1.439904148	3.47E-16	7.93E-15
MIR1915HG	0.337456818	0.852817659	1.337534408	4.36E-11	3.31E-10
AC108463.2	0.038431818	0.156891071	2.029390116	5.66E-10	3.45E-09
AC007038.1	0.140020455	0.37707123	1.429199485	5.04E-10	3.14E-09
AC006033.2	0.0834	0.168336905	1.013232207	0.000144721	0.000245221
LINC02576	0.064477273	0.184184722	1.514290771	0.000506855	0.000770813
AC019171.1	0.014209091	0.159129365	3.48531393	0.008303632	0.009740799
AC232271.1	0.121768182	0.333202579	1.452262364	2.18E-09	1.12E-08
C1RL-AS1	0.192170455	0.608530357	1.662942691	1.95E-16	4.64E-15
FOXD3-AS1	0.240468182	1.104500794	2.199476538	1.08E-06	3.01E-06
DDX11-AS1	0.048222727	0.202654365	2.0712361	3.96E-14	6.40E-13
SNHG12	1.121270455	2.597389286	1.211927952	3.32E-07	1.07E-06
AL035461.2	0.291379545	0.908136111	1.640008939	9.56E-10	5.52E-09
LINC02300	0.196470455	0.096700992	−1.022709779	3.50E-05	6.83E-05
AC012378.2	0.283893182	0.099586111	−1.511331746	0.000152726	0.000257198
AC046143.2	0.791984091	1.787691667	1.174554573	1.18E-09	6.63E-09
AL021707.2	0.106756818	0.233105952	1.126657632	2.69E-10	1.76E-09
AC091563.1	6.838147727	1.15007877	−2.571872914	1.82E-18	8.33E-17
LINC01394	0.056895455	0.117715675	1.048921134	0.000951303	0.001378008
SCAT2	0.0566	0.518727778	3.19610367	1.82E-18	8.33E-17
FOXP4-AS1	0.08025	0.311725992	1.957705156	3.96E-05	7.61E-05
ANKRD10-IT1	1.248854545	2.635794643	1.077632518	4.06E-07	1.26E-06
AC010731.1	0.037329545	0.300609921	3.009502775	0.000307768	0.000486617
C10orf55	0.146629545	0.465756349	1.667399604	1.62E-14	2.97E-13
C10orf71-AS1	1.606786364	0.096897222	−4.051579004	5.90E-05	0.000107582
AC010542.5	0.218320455	0.584991865	1.421969259	1.38E-07	4.84E-07
AP001434.1	0.1947	0.455114484	1.224976617	6.55E-08	2.43E-07
LINC02446	0.264727273	1.053448016	1.992540387	0.00273655	0.00364652
SOCAR	0.04365	0.130051786	1.57503265	2.26E-11	1.94E-10
AL645608.8	0.034854545	0.130198413	1.901293142	4.85E-07	1.46E-06
ATP2A1-AS1	0.272731818	0.64694127	1.246151727	2.48E-05	5.12E-05
AC011462.4	0.119988636	0.378892063	1.65888914	1.88E-07	6.50E-07
AC080129.2	0.062531818	0.166564484	1.413418443	1.02E-05	2.30E-05
AC092119.2	0.046556818	0.23409127	2.330006763	1.19E-12	1.28E-11
C9orf163	0.036684091	0.122457937	1.73905984	6.15E-12	6.14E-11
LINC02528	0.014938636	0.111401984	2.898654562	2.97E-07	9.76E-07
ZNF687-AS1	0.065427273	0.165705952	1.340661387	1.95E-08	7.82E-08
AC015911.3	0.121102273	0.331142262	1.451225206	0.000128232	0.000218632
MMP2-AS1	0.032843182	0.138817262	2.079521172	1.95E-05	4.18E-05
AL031673.1	0.315711364	0.699334127	1.14737572	0.000184101	0.000306276
AL049637.1	0.013915909	0.290593056	4.384193162	2.01E-07	6.85E-07
AC106820.4	0.035827273	0.159904563	2.158080981	0.003041194	0.003984762
AC009951.6	0.251395455	0.112788492	−1.156338689	4.12E-05	7.86E-05
AC004241.3	0.426445455	0.872710913	1.033142615	5.84E-09	2.74E-08
MNX1-AS1	0.091102273	0.435171429	2.256024888	4.74E-07	1.43E-06
LINC01096	0.034438636	0.23257996	2.755626872	1.10E-12	1.21E-11
AC015922.2	4.938929545	2.238169841	−1.141878871	5.18E-05	9.55E-05
AC019080.3	0.115770455	0.286622024	1.307882355	0.006098055	0.007390358
AC012676.1	0.126654545	0.336173016	1.408305072	1.54E-13	2.06E-12
AC092171.4	0.059275	0.225826786	1.929720954	8.04E-09	3.59E-08
AL391056.1	0.06725	0.249414286	1.890937928	0.001803092	0.002493444
ZNF710-AS1	4.998656818	1.293739484	−1.949993346	8.31E-14	1.25E-12
AC087672.1	1.079738636	0.068438492	−3.97973035	2.50E-05	5.14E-05
LINC02362	0.099702273	0.210034921	1.074930915	0.00023382	0.000382046
AL731563.3	0.069831818	0.147256151	1.076371455	1.14E-09	6.54E-09
MIR1-1HG	4.555790909	0.388436706	−3.551950092	6.43E-05	0.000115177
AC234775.2	0.468418182	0.18745	−1.32129125	0.036141895	0.037087664
AC100803.2	0.536195455	0.087641071	−2.613079963	0.018658379	0.020324306
AC012073.1	0.321743182	1.186475198	1.882700462	1.14E-21	1.57E-19
TMPO-AS1	0.351984091	1.246804762	1.824653442	1.57E-16	4.10E-15
AC026310.1	0.011456818	0.219082738	4.257197481	2.57E-14	4.40E-13
PSPC1-AS2	0.141054545	0.328321429	1.218855756	7.14E-07	2.03E-06

## References

[1] Sung, H., Ferlay, J., Siegel, R. L., Laversanne, M., Soerjomataram, I. et al. (2021). Global cancer statistics 2020: GLOBOCAN estimates of incidence and mortality worldwide for 36 cancers in 185 countries. CA: A Cancer Journal for Clinicians*,* 71 *(*3*),* 209–249. 10.3322/caac.21660; 33538338

[2] Kozłowska, J., Kolenda, T., Poter, P., Sobocińska, J., Guglas, K. et al. (2021). Long intergenic non-coding RNAs in HNSCC: From “Junk DNA” to important prognostic factor. Cancers*,* 13 *(*12*),* 2949. 10.3390/cancers13122949; 34204634 PMC8231241

[3] Johnson, D. E., Burtness, B., Leemans, C. R., Lui, V. W. Y., Bauman, J. E. et al. (2020). Head and neck squamous cell carcinoma. Nature Reviews Disease Primers*,* 6 *(*1*),* 92. 10.1038/s41572-020-00224-3; 33243986 PMC7944998

[4] Goel, B., Tiwari, A. K., Pandey, R. K., Singh, A. P., Kumar, S. et al. (2022). Therapeutic approaches for the treatment of head and neck squamous cell carcinoma—An update on clinical trials. Translational Oncology*,* 21 *(*3*),* 101426. 10.1016/j.tranon.2022.101426; 35460943 PMC9046875

[5] Lee, S., Schmitt, C. A. (2019). The dynamic nature of senescence in cancer. Nature Cell Biology*,* 21 *(*1*),* 94–101. 10.1038/s41556-018-0249-2; 30602768

[6] Montes, M., Lubas, M., Arendrup, F. S., Mentz, B., Rohatgi, N. et al. (2021). The long non-coding RNA MIR31HG regulates the senescence associated secretory phenotype. Nature Communications*,* 12 *(*1*),* 2459. 10.1038/s41467-021-22746-4; 33911076 PMC8080841

[7] Stoczynska-Fidelus, E., Węgierska, M., Kierasińska, A., Ciunowicz, D., Rieske, P. (2022). Role of senescence in tumorigenesis and anticancer therapy. Journal of Oncology*,* 2022*,* 5969536. 10.1155/2022/5969536; 35342397 PMC8956409

[8] Zhai, W. Y., Duan, F. F., Chen, S., Wang, J. Y., Zhao, Z. R. et al. (2022). An aging-related gene signature-based model for risk stratification and prognosis prediction in lung squamous carcinoma. Frontiers in Cell and Developmental Biology*,* 10*,* 770550. 10.3389/fcell.2022.770550; 35300428 PMC8921527

[9] Yang, J., Jiang, Q., Liu, L., Peng, H., Wang, Y. et al. (2020). Identification of prognostic aging-related genes associated with immunosuppression and inflammation in head and neck squamous cell carcinoma. Sedentary Life and Nutrition*,* 12 *(*24*),* 25778–25804. 10.18632/aging.104199; 33232279 PMC7803584

[10] Beylerli, O., Gareev, I., Sufianov, A., Ilyasova, T., Guang, Y. (2022). Long noncoding RNAs as promising biomarkers in cancer. Non-Coding RNA Research*,* 7 *(*2*),* 66–70. 10.1016/j.ncrna.2022.02.004; 35310927 PMC8891810

[11] Zhao, Z., Liu, H., Zhou, X., Fang, D., Ou, X. et al. (2021). Necroptosis-related lncRNAs: Predicting prognosis and the distinction between the cold and hot tumors in gastric cancer. Journal of Oncology*,* 2021*,* 6718443. 10.1155/2021/6718443; 34790235 PMC8592775

[12] Luo, L., Li, L., Liu, L., Feng, Z., Zeng, Q. et al. (2022). A necroptosis-related lncRNA-based signature to predict prognosis and probe molecular characteristics of stomach adenocarcinoma. Frontiers in Genetics*,* 13*,* 833928. 10.3389/fgene.2022.833928; 35330731 PMC8940523

[13] Robin, X., Turck, N., Hainard, A., Tiberti, N., Lisacek, F. et al. (2011). pROC: An open-source package for R and S+ to analyze and compare ROC curves. BMC Bioinformatics*,* 12 *(*1*),* 77. 10.1186/1471-2105-12-77; 21414208 PMC3068975

[14] Yu, G., Wang, L. G., Han, Y., He, Q. Y. (2012). clusterProfiler: An R package for comparing biological themes among gene clusters. OMICS: A Journal of Integrative Biology*,* 16 *(*5*),* 284–287. 10.1089/omi.2011.0118; 22455463 PMC3339379

[15] Zheng, A., Xie, F., Shi, S., Liu, S., Long, J. et al. (2022). Sustained drug release from liposomes for the remodeling of systemic immune homeostasis and the tumor microenvironment. Frontiers in Immunology*,* 13*,* 829391. 10.3389/fimmu.2022.829391; 35493504 PMC9039229

[16] Tettamanti, S., Rotiroti, M. C., Giordano Attianese, G. M. P., Arcangeli, S., Zhang, R. et al. (2022). Lenalidomide enhances CD23.CAR T cell therapy in chronic lymphocytic leukemia. Leukemia and Lymphoma*,* 63 *(*7*),* 1566–1579. 10.1080/10428194.2022.2043299; 35259043 PMC9828187

[17] Gong, W., Wang, L., Schubert, M. L., Kleist, C., Neuber, B. et al. (2022). HDAC inhibition for optimized cellular immunotherapy of NY-ESO-1-positive soft tissue sarcoma. Biomedicines*,* 10 *(*2*),* 373. 10.3390/biomedicines10020373; 35203582 PMC8962361

[18] Wang, H., Chen, H., Liu, S., Zhang, J., Lu, H. et al. (2021). Costimulation of γδTCR and TLR7/8 promotes Vδ2 T-cell antitumor activity by modulating mTOR pathway and APC function. Journal for ImmunoTherapy of Cancer*,* 9 *(*12*),* e003339. 10.1136/jitc-2021-003339; 34937742 PMC8705233

[19] Veneziani, I., Infante, P., Ferretti, E., Melaiu, O., Battistelli, C. et al. (2021). Nutlin-3a enhances natural killer cell-mediated killing of neuroblastoma by restoring p53-dependent expression of ligands for NKG2D and DNAM-1 receptors. Cancer Immunology Research*,* 9 *(*2*),* 170–183. 10.1158/2326-6066.CIR-20-0313; 33303573

[20] Li, J., Zhou, S., Yu, J., Cai, W., Yang, Y. et al. (2021). Low dose shikonin and anthracyclines coloaded liposomes induce robust immunogenetic cell death for synergistic chemo-immunotherapy. Journal of Controlled Release*,* 335*,* 306–319. 10.1016/j.jconrel.2021.05.040; 34081995

[21] Chung, Y. M., Khan, P. P., Wang, H., Tsai, W. B., Qiao, Y. et al. (2021). Sensitizing tumors to anti-PD-1 therapy by promoting NK and CD8+ T cells via pharmacological activation of FOXO3. Journal for ImmunoTherapy of Cancer*,* 9 *(*12*),* e002772. 10.1136/jitc-2021-002772; 34887262 PMC8663085

[22] Marks, D. K., Gartrell, R. D., El Asmar, M., Boboila, S., Hart, T. et al. (2020). Akt inhibition is associated with favorable immune profile changes within the tumor microenvironment of hormone receptor positive, HER2 negative breast cancer. Frontiers in Oncology*,* 10*,* 968. 10.3389/fonc.2020.00968; 32612958 PMC7308467

[23] Liu, G., Jin, Z., Lu, X. (2020). Differential targeting of Gr-MDSCs, T cells and prostate cancer cells by dactolisib and dasatinib. International Journal of Molecular Sciences*,* 21 *(*7*),* 2337. 10.3390/ijms21072337; 32230980 PMC7178187

[24] Kobayashi, Y., Yamada, D., Kawai, T., Sato, Y., Teshima, T. et al. (2020). Different immunological effects of the molecular targeted agents sunitinib, everolimus and temsirolimus in patients with renal cell carcinoma. International Journal of Oncology*,* 56 *(*4*),* 999–1013. 10.3892/ijo.2020.4975; 32319571

[25] Ge, M., Hu, Z., Chen, X., Du, G., Wang, H. et al. (2019). PCC0208018 exerts antitumor effects by activating effector T cells. International Journal of Immunopathology and Pharmacology*,* 33*,* 2058738419843366. 10.1177/2058738419843366; 30968715 PMC6458668

[26] Ferrari de Andrade, L., Ngiow, S. F., Stannard, K., Rusakiewicz, S., Kalimutho, M. et al. (2014). Natural killer cells are essential for the ability of BRAF inhibitors to control BRAFV600E-mutant metastatic melanoma. Cancer Research*,* 74 *(*24*),* 7298–7308. 10.1158/0008-5472.CAN-14-1339; 25351955

[27] Onishi, H., Morisaki, T., Kiyota, A., Koya, N., Tanaka, H. et al. (2013). The Hedgehog inhibitor cyclopamine impairs the benefits of immunotherapy with activated T and NK lymphocytes derived from patients with advanced cancer. Cancer Immunology, Immunotherapy*,* 62 *(*6*),* 1029–1039. 10.1007/s00262-013-1419-5; 23591983 PMC11029486

[28] Bridle, B. W., Chen, L., Lemay, C. G., Diallo, J. S., Pol, J. et al. (2013). HDAC inhibition suppresses primary immune responses, enhances secondary immune responses, and abrogates autoimmunity during tumor immunotherapy. Molecular Therapy*,* 21 *(*4*),* 887–894. 10.1038/mt.2012.265; 23295947 PMC3616544

[29] Jiang, Q., Weiss, J. M., Back, T., Chan, T., Ortaldo, J. R. et al. (2011). mTOR kinase inhibitor AZD8055 enhances the immunotherapeutic activity of an agonist CD40 antibody in cancer treatment. Cancer Research*,* 71 *(*12*),* 4074–4084. 10.1158/0008-5472.CAN-10-3968; 21540234 PMC3116937

[30] de Billy, E., Pellegrino, M., Orlando, D., Pericoli, G., Ferretti, R. et al. (2021). Dual IGF1R/IR inhibitors in combination with GD2-CAR T-cells display a potent anti-tumor activity in diffuse midline glioma H3K27M-mutant. Neuro-Oncology*,* 24 *(*7*),* 1150–1163. 10.1093/neuonc/noab300; 34964902 PMC9248389

[31] Hardman, C., Ho, S., Shimizu, A., Luu-Nguyen, Q., Sloane, J. L. et al. (2020). Synthesis and evaluation of designed PKC modulators for enhanced cancer immunotherapy. Nature Communications*,* 11 *(*1*),* 1879. 10.1038/s41467-020-15742-7; 32312992 PMC7170889

[32] Takai, S., Sabzevari, H., Farsaci, B., Schlom, J., Greiner, J. W. (2012). Distinct effects of saracatinib on memory CD8+ T cell differentiation. Journal of Immunology*,* 188 *(*9*),* 4323–4333. 10.4049/jimmunol.1101439; 22450814 PMC3378668

[33] Powell, S. F., Gold, K. A., Gitau, M. M., Sumey, C. J., Lohr, M. M. et al. (2020). Safety and efficacy of pembrolizumab with chemoradiotherapy in locally advanced head and neck squamous cell carcinoma: A phase IB study. Journal of Clinical Oncology*,* 38 *(*21*),* 2427–2437. 10.1200/JCO.19.03156; 32479189 PMC7365766

[34] Bauman, J. E., Ohr, J., Gooding, W. E., Ferris, R. L., Duvvuri, U. et al. (2020). Phase I study of ficlatuzumab and cetuximab in cetuximab-resistant, recurrent/metastatic head and neck cancer. Cancers*,* 12 *(*6*),* 1537. 10.3390/cancers12061537; 32545260 PMC7352434

[35] Akbari Dilmaghani, N., Khoshsirat, S., Shanaki-Bavarsad, M., Pourbagheri-Sigaroodi, A., Bashash, D. (2021). The contributory role of long non-coding RNAs (lncRNAs) in head and neck cancers: Possible biomarkers and therapeutic targets? European Journal of Pharmacology*,* 900 *(*3*),* 174053. 10.1016/j.ejphar.2021.174053; 33766619

[36] Morgan, E. L., Chen, Z., van Waes, C. (2020). Regulation of NFκB signalling by ubiquitination: A potential therapeutic target in head and neck squamous cell carcinoma? Cancers*,* 12 *(*10*),* 2877. 10.3390/cancers12102877; 33036368 PMC7601648

[37] Sailer, V., Gevensleben, H., Dietrich, J., Goltz, D., Kristiansen, G. et al. (2017). Clinical performance validation of PITX2 DNA methylation as prognostic biomarker in patients with head and neck squamous cell carcinoma. PLoS One*,* 12 *(*6*),* e0179412. 10.1371/journal.pone.0179412; 28617833 PMC5472307

[38] Lin, M. C., Huang, M. C., Lou, P. J. (2021). Anti-C1GALT1 autoantibody is a novel prognostic biomarker for patients with head and neck cancer. Laryngoscope*,* 131 *(*1*),* E196–E202. 10.1002/lary.28694; 32427353

[39] Lin, W., Xu, Y., Gao, J., Zhang, H., Sun, Y. et al. (2021). Multi-omics data analyses identify B7-H3 as a novel prognostic biomarker and predict response to immune checkpoint blockade in head and neck squamous cell carcinoma. Frontiers in Immunology*,* 12*,* 757047. 10.3389/fimmu.2021.757047; 34675936 PMC8524082

[40] Kudlova, N., de Sanctis, J. B., Hajduch, M. (2022). Cellular senescence: Molecular targets, biomarkers, and senolytic drugs. International Journal of Molecular Sciences*,* 23 *(*8*),* 4168. 10.3390/ijms23084168; 35456986 PMC9028163

[41] Park, S. S., Choi, Y. W., Kim, J. H., Kim, H. S., Park, T. J. (2021). Senescent tumor cells: An overlooked adversary in the battle against cancer. Experimental and Molecular Medicine*,* 53 *(*12*),* 1834–1841. 10.1038/s12276-021-00717-5; 34916607 PMC8741813

[42] Popov, A., Mandys, V. (2022). Senescence-associated miRNAs and their role in pancreatic cancer. Pathology Oncology Research*,* 28*,* 1610156. 10.3389/pore.2022.1610156; 35570840 PMC9098800

[43] Wang, Z., Li, Y., Wu, D., Yu, S., Wang, Y. et al. (2020). Nuclear receptor HNF4α performs a tumor suppressor function in prostate cancer via its induction of p21-driven cellular senescence. Oncogene*,* 39 *(*7*),* 1572–1589. 10.1038/s41388-019-1080-3; 31695151 PMC7018660

[44] Jia, Y., Jin, H., Gao, L., Yang, X., Wang, F. et al. (2020). A novel lncRNA PLK4 up-regulated by talazoparib represses hepatocellular carcinoma progression by promoting YAP-mediated cell senescence. Journal of Cellular and Molecular Medicine*,* 24 *(*9*),* 5304–5316. 10.1111/jcmm.15186; 32243714 PMC7205816

[45] Zou, J., Ma, Q., Sun, R., Cai, J., Liao, H. et al. (2019). Dihydroartemisinin inhibits HepG2.2.15 proliferation by inducing cellular senescence and autophagy. BMB Reports*,* 52 *(*8*),* 520–524. 10.5483/BMBRep.2019.52.8.058; 31383247 PMC6726210

[46] Lee, Y. H., Chen, Y. Y., Yeh, Y. L., Wang, Y. J., Chen, R. J. (2019). Stilbene compounds inhibit tumor growth by the induction of cellular senescence and the inhibition of telomerase activity. International Journal of Molecular Sciences*,* 20 *(*11*),* 2716. 10.3390/ijms20112716; 31159515 PMC6600253

[47] Li, R., Zhang, X., Tian, X., Shen, C., Zhang, Q. et al. (2017). Triptolide inhibits tumor growth by induction of cellular senescence. Oncology Reports*,* 37 *(*1*),* 442–448. 10.3892/or.2016.5258; 27878302

[48] Geng, R., Song, J., Zhong, Z., Ni, S., Liu, W. et al. (2022). Crosstalk of redox-related subtypes, establishment of a prognostic model and immune responses in endometrial carcinoma. Cancers*,* 14 *(*14*),* 3383. 10.3390/cancers14143383; 35884444 PMC9319597

[49] Hu, Y., Song, J., Wang, Z., Kan, J., Ge, Y. et al. (2021). A novel S100 family-based signature associated with prognosis and immune microenvironment in glioma. Journal of Oncology*,* 2021 *(*2*),* 3586589. 10.1155/2021/3586589; 34712325 PMC8548170

[50] Song, J., Liu, Y., Guan, X., Zhang, X., Yu, W. et al. (2021). A novel ferroptosis-related biomarker signature to predict overall survival of esophageal squamous cell carcinoma. Frontiers in Molecular Biosciences*,* 8*,* 675193. 10.3389/fmolb.2021.675193; 34291083 PMC8287967

[51] Lu, Y., Li, L., Chen, H., Jing, X., Wang, M. et al. (2021). Peroxiredoxin1 knockdown inhibits oral carcinogenesis via inducing cell senescence dependent on mitophagy. OncoTargets and Therapy*,* 14*,* 239–251. 10.2147/OTT.S284182; 33469304 PMC7812030

[52] Lan, Y. Y., Chang, F. H., Tsai, J. H., Chang, Y. (2018). Epstein-Barr virus Rta promotes invasion of bystander tumor cells through paracrine of matrix metalloproteinase 9. Biochemical and Biophysical Research Communications*,* 503 *(*3*),* 2160–2166. 10.1016/j.bbrc.2018.08.006; 30082032

[53] Chen, F., Gong, X., Xia, M., Yu, F., Wu, J. et al. (2022). The aging-related prognostic signature reveals the landscape of the tumor immune microenvironment in head and neck squamous cell carcinoma. Frontiers in Oncology*,* 12*,* 857994. 10.3389/fonc.2022.857994; 35619896 PMC9127417

[54] Gu, H., Song, J., Chen, Y., Wang, Y., Tan, X. et al. (2022). Inflammation-related LncRNAs signature for prognosis and immune response evaluation in uterine corpus endometrial carcinoma. Frontiers in Oncology*,* 12*,* 923641. 10.3389/fonc.2022.923641; 35719911 PMC9201290

[55] Ghafouri-Fard, S., Mohammad-Rahimi, H., Jazaeri, M., Taheri, M. (2020). Expression and function of long non-coding RNAs in head and neck squamous cell carcinoma. Experimental and Molecular Pathology*,* 112 *(*12*),* 104353. 10.1016/j.yexmp.2019.104353; 31812485

[56] Tang, Y., Li, C., Zhang, Y. J., Wu, Z. H. (2021). Ferroptosis-related long non-coding RNA signature predicts the prognosis of Head and neck squamous cell carcinoma. International Journal of Biological Sciences*,* 17 *(*3*),* 702–711. 10.7150/ijbs.55552; 33767582 PMC7975700

[57] Wu, C., Liu, F., Chen, H., Liu, Q., Song, C. et al. (2022). Identification of ferroptosis-related lncRNA pairs for predicting the prognosis of head and neck squamous cell carcinoma. Journal of Oncology*,* 2022*,* 7602482. 10.1155/2022/7602482; 35909900 PMC9328971

[58] Jiang, W., Song, Y., Zhong, Z., Gao, J., Meng, X. (2021). Ferroptosis-related long non-coding RNA signature contributes to the prediction of prognosis outcomes in head and neck squamous cell carcinomas. Frontiers in Genetics*,* 12*,* 785839. 10.3389/fgene.2021.785839; 34976018 PMC8718757

[59] Qiu, L., Tao, A., Liu, F., Ge, X., Li, C. (2022). Potential prognostic value of a eight ferroptosis-related lncRNAs model and the correlative immune activity in oral squamous cell carcinoma. BMC Genomic Data*,* 23 *(*1*),* 80. 10.1186/s12863-022-01097-z; 36384476 PMC9667687

[60] Xiao, L., Shi, X. Y., Li, Z. L., Li, M., Zhang, M. M. et al. (2021). Downregulation of LINC01508 contributes to cisplatin resistance in ovarian cancer via the regulation of the Hippo-YAP pathway. Journal of Gynecologic Oncology*,* 32 *(*5*),* e77. 10.3802/jgo.2021.32.e77; 34132072 PMC8362814

[61] Liu, H., Zhang, L., Ding, X., Sui, X. (2021). LINC00861 inhibits the progression of cervical cancer cells by functioning as a ceRNA for miR‐513b‐5p and regulating the PTEN/AKT/mTOR signaling pathway. Molecular Medicine Reports*,* 23 *(*1*),* 24. 10.3892/mmr.2020.11662; 33179755 PMC7673320

[62] Maggiorani, D., Beauséjour, C. (2021). Senescence and aging: Does it impact cancer immunotherapies? Cells*,* 10 *(*7*),* 1568. 10.3390/cells10071568; 34206425 PMC8307798

[63] Faget, D. V., Ren, Q., Stewart, S. A. (2019). Unmasking senescence: Context-dependent effects of SASP in cancer. Nature Reviews: Cancer*,* 19 *(*8*),* 439–453. 10.1038/s41568-019-0156-2; 31235879

[64] Chambers, C. R., Ritchie, S., Pereira, B. A., Timpson, P. (2021). Overcoming the senescence-associated secretory phenotype (SASP): A complex mechanism of resistance in the treatment of cancer. Molecular Oncology*,* 15 *(*12*),* 3242–3255. 10.1002/1878-0261.13042; 34137158 PMC8637570

[65] Lin, W., Chen, M., Hong, L., Zhao, H., Chen, Q. (2018). Crosstalk between PD-1/PD-L1 blockade and its combinatorial therapies in tumor immune microenvironment: A focus on HNSCC. Frontiers in Oncology*,* 8*,* 532. 10.3389/fonc.2018.00532; 30519541 PMC6258806

[66] Hanoteau, A., Newton, J. M., Krupar, R., Huang, C., Liu, H. C. et al. (2019). Tumor microenvironment modulation enhances immunologic benefit of chemoradiotherapy. Journal for ImmunoTherapy of Cancer*,* 7 *(*1*),* 10. 10.1186/s40425-018-0485-9; 30646957 PMC6332704

[67] Toso, A., Revandkar, A., di Mitri, D., Guccini, I., Proietti, M. et al. (2014). Enhancing chemotherapy efficacy in Pten-deficient prostate tumors by activating the senescence-associated antitumor immunity. Cell Reports*,* 9 *(*1*),* 75–89. 10.1016/j.celrep.2014.08.044; 25263564

